# CAPN2 promotes apalutamide resistance in metastatic hormone-sensitive prostate cancer by activating protective autophagy

**DOI:** 10.1186/s12967-024-05335-z

**Published:** 2024-06-06

**Authors:** Zihao Qi, Xiaojie Bai, Linjie Wu, Peng Zhang, Zhongqiang Guo, Ying Yu

**Affiliations:** 1 Department of Urology, Zhongnan Hospital of Wuhan University, Wuhan, 430071 P. R. China; 2 Cancer Precision Diagnosis and Treatment and Translational Medicine, Hubei Engineering Research Center, Zhongnan Hospital of Wuhan University, Wuhan, 430071 P. R. China; 3 Huaihe Hospital of Henan University, Kaifeng, 475000 P. R. China

**Keywords:** Calpain 2, Forkhead Box O1, Apalutamide, Autophagy, Prostate cancer

## Abstract

Apalutamide, a novel endocrine therapy agent, has been shown to significantly improve the prognosis of patients with metastatic hormone-sensitive prostate cancer (mHSPC). However, resistance to apalutamide has also been reported, and the underlying mechanism for this response has yet to be clearly elucidated. First, this study established apalutamide-resistant prostate cancer (PCa) cells, and confirmed that apalutamide activated the release of calcium ions (Ca^2+^) and endoplasmic reticulum stress (ERS) to enhance autophagy. Second, RNA sequencing, western blotting, and immunohistochemistry revealed significantly decreased Calpain 2 (CAPN2) expression in the apalutamide-resistant PCa cells and tissues. Furthermore, immunofluorescence and transmission electron microscopy (TEM) showed that CAPN2 promoted apalutamide resistance by activating protective autophagy. CAPN2 promoted autophagy by reducing Forkhead Box O1 (FOXO1) degradation while increasing nuclear translocation via nucleoplasmic protein isolation and immunofluorescence. In addition, FOXO1 promoted protective autophagy through the transcriptional regulation of autophagy-related gene 5 (ATG5). Furthermore, a dual-fluorescence assay confirmed that transcription factor 3 (ATF3) stimulation promoted CAPN2-mediated autophagy activation via transcriptional regulation. In summary, CAPN2 activated protective autophagy by inhibiting FOXO1 degradation and promoting its nuclear translocation via transcriptional ATG5 regulation. ATF3 activation and transcriptional CAPN2 regulation jointly promoted this bioeffect. Thus, our findings have not only revealed the mechanism underlying apalutamide resistance, but also provided a promising new target for the treatment of metastatic PCa.

**Supplementary Information:**

The online version contains supplementary material available at 10.1186/s12967-024-05335-z.

## Introduction

Androgen deprivation therapy (ADT) is a standard approach for supporting male patients with metastatic hormone-sensitive prostate cancer (mHSPC), typically providing a 60–80% response rate after two years of treatment. However, there are numerous reports of patients subsequently acquiring castration-resistant prostate cancer (CRPC), which is a leading cause of death. Notably, the five-year relative survival rate of mHSPC is approximately 30%, while the length of metastatic CRPC (mCRPC) median survival is significantly shorter at approximately three years [1]. Apalutamide inhibits the ligand-binding domain of the androgen receptor (AR) and has been approved for treating mHSPC and nonmetastatic CRPC (nmCRPC) [2]. The TITAN trial (in which 1052 patients with mHSPC randomly received ADT either with or without apalutamide) clearly demonstrated that apalutamide improved overall survival (OS) at a median follow-up of two years [3]. However, since acquired resistance to these second-generation AR antagonists remains a major challenge during advanced prostate cancer (PCa) treatment, it is essential to determine the underlying mechanisms involved in this resistance [4, 5].

Autophagy, an adaptive process facilitating the turnover of long-lived and toxic macromolecules as well as organelles, plays a crucial dual role in the progression of various diseases and in maintaining physiological homeostasis. In cancers specifically, autophagy can help prevent early tumor development and the metabolic adaptation of metastatic tumors [6]. In this close association between autophagy and apoptosis, autophagy has been proven to affect PCa therapy responses [7]. Our earlier research showed that the activation of protective autophagy promoted metastasis in PCa [8], and δ-tocotrienol exhibited anti-cancer activity in CRPC by triggering ERS-mediated autophagy [9]. In addition, ERS is often accompanied by the opening of Ca^2+^ channels and higher intracellular Ca^2+^ concentrations, facilitating a series of functional changes. Based on these results, it is reasonable to assume that autophagy plays an important role in apalutamide resistance and may be significantly promoted by the ERS activated by apalutamide. However, the specific mechanisms by which protective autophagy regulates mHSPC apalutamide resistance and the role of apalutamide-mediated ERS activation remain unclear.

Therefore, this study constructs apalutamide-resistant PCa cells and identifies differentially expressed genes (DEGs) via RNA sequencing and bioinformatics analysis. Furthermore, low levels of CAPN2, a calcium-regulated non-lysosomal mercaptan protease, promote apalutamide-resistance by mediating ERS and cellular autophagy. CAPN2 is known for catalyzing the limited proteolysis of substrates critical for cytoskeletal remodeling and signal transduction. During intracellular calcium accumulation in the process of ERS, the phosphorylation of calcium/calmodulin-dependent protein kinase II (CAMK II) activates CAPN2, after which it plays a critical role in autophagy [10]. These findings confirm that CAPN2 functionality is largely regulated by ERS and intracellular calcium levels. The overexpression of CAPN2 has been found to accelerate tumor progression in numerous malignancies and is, thus, associated with poor prognosis [11]. Furthermore, an in vitro study has shown that the combination of CAPN2 inhibitor and ADT may provide a promising therapeutic strategy for the prevention of PCa progression [12]. Our study aimed to reveal the mechanism underlying the promotion of apalutamide resistance in mHSPC via CAPN2-mediated protective autophagy.

## Materials and methods

### Cell lines and treatment

Classical human prostate cancer cell lines (PC-3, DU145, C4-2 and LNCaP) were obtained from the Institute of Biochemistry and Cell Biology of Chinese Academy of Sciences (Shanghai, P.R. China). All the cell lines were maintained in RPMI 1640 medium supplemented with 10% fetal bovine serum. Immortalized human prostatic epithelial cell line RWPE-1 was obtained from Prof. Xiantao Zeng (Zhongnan Hospital, Wuhan, China) and grown in PEpiCM without FBS (ScienCell, USA). All cells were cultured at 37℃ with 5% CO_2_ and 95% humidified air, and the culture media was supplemented with 1% penicillin/streptomycin (P/S, 15140-122, Gibco).

#### Construction of apalutamide-resistant PCa cell lines

AR positive prostate cancer cell lines LNCaP and C4-2 were cultured in the presence of increasing doses of apalutamide. Drug treatment was started using 0.2µM apalutamide. Drug-containing medium was changed every two days. Cells were splitted when 80% confluency was reached. Apalutamide concentration was increased when cells started to regrow in the presence of the drug at a growth rate similar to that of control cells until final concentrations (LNCaP: 45.48µM; C4-2: 20.16µM) were reached. Cells were then cultured for another 8 weeks in these conditions.

### RNA extraction and qRT-PCR assay

Total RNA was isolated using Hipure Total RNA Mini Kit (Magen, R4111-03). 1 µg of total RNA was subjected to reverse transcription into cDNA using Hiscript III RT SuperMix (Vazyme, R323-01). Quantitative real-time PCR (qRT-PCR) was conducted using a QuantStudio 1 system (Thermo Fisher Scientific) with SYBR qPCR Master Mix (Vazyme, Q711-02). The expression level of specific genes was normalized to the expression level of GAPDH using the comparative CT method (2^−ΔΔCT^). The primer sequences are presented in Supplementary Table 1.

### RNA sequencing

Total RNA was isolated from apalutamide-resistant LNCaP and C4-2 cells and the corresponding control cells using RNeasy Mini kit (Qiagen). Transcriptome sequencing on an Illumina HiSeq X Ten platform was carried out by BIOMARKER Tech (Wuhan, China).

### Plasmid transfections

Three types of plasmids were constructed by Vigene Biosciences (Shangdong, China) and Paivi Biosciences (Wuhan, China) including OE-CAPN2 plasmids, Si-ATF3plasmids. Plasmids were transfected into LNCaP and C4-2 cells by using Lipofectamine 2000 (Invitrogen) according to manufacturer’s instruction.

### Western blotting

Cellular protein was extracted using RIPA Lysis Buffer (Thermo Fisher Scientifific) according to the manufacturer’s instructions. The concentration of total protein was measured by BCA protein assay kit (Beyotime, P0012). Total protein was separated by 10% SDS-PAGE gels and transferred onto polyvinylidene diflfluoride membranes (Millipore, IPVH00010) after electrophoresis was completed. Membranes were blocked by 5% nonfat milk before incubated with primary antibodies overnight at 4℃ followed by hybridized with specifific horseradish peroxidase (HRP)-conjugated secondary antibody (Proteintech) at room temperature for 1 h and visualized using ECL system (BIO-RAD). Detailed information regarding the primary antibodies used in this study is listed in Supplementary Table 2.

### Cell counting Kit-8 (CCK-8) assay

The cells were seeded into 96-well plates at 5000 cells per well and cultured for 24 h. Then, 10% CCK-8 (Dojindo Laboratories, CK04) medium were added into each well before the plates were incubated for another hour. The absorbance was measured at 450 nm with a SpectraMax M5 microplate reader (MD, USA). This process was repeated for 5 days. The cell doubling times were calculated using GraphPad Prism 7.0 software (La Jolla, USA), while the OD values were used to perform the statistical analysis.

### Transwell migration assay

The cell migration was evaluated using a 24-well Transwell plate with 8.0-mm pore polycarbonate membrane inserts (Corning). Homogeneous single-cell suspensions (200 µl; 1 × 10^5^ cells/well) in serum-free mediums were added to the upper chambers, while 500 ml complete mediums were added to the lower chambers. After incubation for 24 h at 37 ℃ in a CO2 incubator, the migrated or invaded cells were fixed with ice-cold methanol and stained with 0.1% crystal violet for 15 min at room temperature. The migrated cells were counted in three randomly chosen fields using an inverted phase-contrast microscope (Olympus, Tokyo, Japan) at 100× magnification.

### Colony formation assay

C4-2 and LNCaP and the corresponding parental cells were grown in a 6-well plate using a cell density of 1000 cells per well. Then, the cells were fixed using 4% paraformaldehyde for 15 min prior to be stained with 0.5% crystal violet solution for 30 min. The number of colonies (> 50 cells) were counted under a microscope.

### Flow cytometry analysis

For cell cycle analysis, cells were harvested and fixed in 70% prechilled ethanol at -20℃ for 4 h. Then, after washed twice with PBS, the fixed cells were stained with propidium iodide (PI) buffer (Servicebio, G1021) for 30 min. For apoptosis analysis, the cells were incubated with annexin V-FITC and PI solutions (Vazyme, A211-02) for 20 min. The apoptosis analysis was performed by Cytoflex S flow cytometer.

### Transmission electron microscopy (TEM)

The cells were fixed with 2.5% glutaraldehyde and 1% osmium tetroxide at 4℃ for 2 h and dehydrated before immersed in spur resin. Then the cells were cut into 50 nm sections and stained with 4% uranyl acetate and lead citrate. The images of mitochondria in PCa cells were captured with the HT7700 transmission electron microscope (Hitachi, Japan).

### Immunofluorescence staining assay

The cells were fixed with 4% paraformaldehyde for 15 min and permeabilized with 0.5% Triton X-100 for 10 min. Afterward, 5% donkey serum in PBS was used to block non-specific binding. Then, the cells were incubated with primary antibodies overnight at 4℃ and with secondary antibody CoraLite 488-conjugated anti-rabbit IgG (Proteintech, SA00013-2) or CoraLite 594-conjugated anti-mouse IgG (Proteintech, SA00013-3) for 2 h at room temperature in the dark. The cell nuclei were stained with DAPI (Servicebio, G1012) at room temperature for 6 min. the data were analyzed using a Nikon A1Si laser scanning confocal microscope (Nikon Instruments Inc., Japan). Detailed information regarding drugs and reagents used in this study is listed in Supplementary Table 3.

### Measurement of intracellular calcium ion (Ca^2+^)

After the cells were fixed with 4% paraformaldehyde for 10 min, the cells were incubated with Fluo-4 AM (MCE, HY-101,896)(10µM/ml) in dark at 37 °C for 30 min. Next, the cell nucleus was stained in a dark for 10 min using DAPI containing quenched sealer tablets. Then, the absorbance value at 488 nm was measured using a microplate reader. Nikon A1Si laser scanning confocal microscope was used to analyze the data.

### Luciferase reporter assay

Luciferase reporter assay was performed to test the transcriptional regulation of ATF3 on CAPN2. LNCaP or C4-2 cells were co-transfected with overexpressed ATF3 plasmids, CAPN2 luciferase reporter plasmid (pGL3-basic plasmids containing promoter of CAPN2) and pRL-TK renilla luciferase reporter vectors. A total of 48 h later, the activities of firefly luciferase and renilla luciferase were detected using the Dual-Luciferase Reporter Assay System (Promega) according to manufacturer’s protocol. The sequences of ATF3 and CAPN2 binding sites are shown in Supplementary Tables 4– 5.

### Tumor xenograft assay

To investigate the role of CAPN2 in subcutaneous tumor formation and lung metastasis in PCa, we chose 4-week-old female BALB/c nude mice for tumor xenograft experiments, which randomly were divided into four groups (*n* = 5 per group). 3 × 10^6^ PCa cells with Cy3 (LNCaP-NC; LNCaP-OE-CAPN2) were subcutaneously injected into the right axilla and tail vain of the nude mice. The tumor volume and weight of mice were monitored and recorded. Specimens were collected six weeks after the experiment. Tumor volume was calculated according to the formula (Tumor volume = π/6 × length × width^2^). The In Vivo FX PRO (BRUKER Corporation, USA) was used to obtain fluorescence images of xenografts in nude mice. All animal experiments were allowed in the light of NIH Guidelines for the Care and Use of Laboratory Animals and approved by the Animal Care Committee of Zhongnan hospital of Wuhan university.

### Statistical analysis

All data were presented as mean SD. Analysis was performed using GraphPad Prism 7.0 software. Student t-test was used to evaluate the group difference. Kaplan–Meier survival analysis and log-rank test were used to assess survival difference. All statistical tests were considered statistically significant when P values less than 0.05.

## Results

### Apalutamide-resistant PCa cells are characterized by protective autophagy and increased metastatic potential

**Fig. 1:**
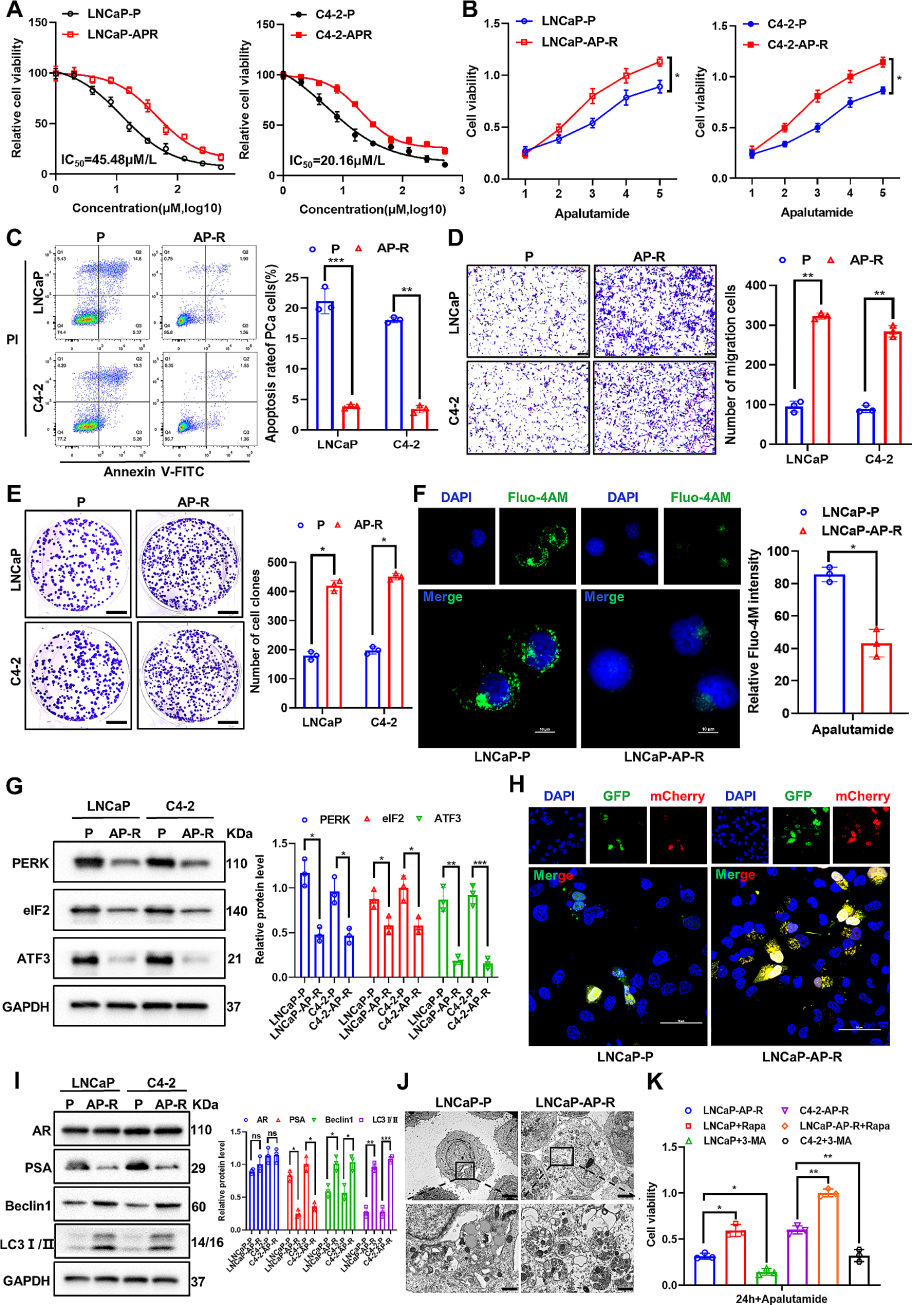
Apalutamide-resistant PCa cells are characterized by protective autophagy and increased metastatic potential. (**A**) The IC_50_ value of apalutamide was measured in LNCaP-P/AP-R and C4-2-P/AP-R cells by CCK-8 assay. (**B**) The cell survival curve of LNCaP-P/AP-R and C4-2-P/AP-R cells was detected by CCK-8 assay. (**C**) The apoptosis rate of LNCaP-P/AP-R and C4-2-P/AP-R cells was detected by flow cytometry assay. (**D**) The cell migration ability of LNCaP-P/AP-R and C4-2-P/AP-R cells was confirmed by tanswell experiment. (**E**) The proliferative ability of LNCaP-P/AP-R and C4-2-P/AP-R cells was confirmed by plate clone formation assay. (**F**) The level of intracellular calcium ions (Fluo-4 AM) in LNCaP-P/AP-R and C4-2-P/AP-R cells was confirmed by immunofluorescence assay. (**G**) Endoplasmic reticulum stress-related proteins expression in LNCaP-P/AP-R and C4-2-P/AP-R cells were detected by western blot assay. (**H**) The level of autophagy flow in LNCaP-P/AP-R cells was confirmed by immunofluorescence assay. (**I**) The autophagy related proteins in LNCaP-P/AP-R and C4-2-P/AP-R cells were detected by western blot assay. (**J**) The morphology, structure and number of autophagosomes was confirmed by TEM. (**K**) The survival rate after the addition of an autophagy activator (Rapa) or an autophagy inhibitor (3-MA) to LNCaP-AP-R and C4-2-AP-R cells, respectively, was confirmed by CCK-8 assay

Apalutamide is a fairly new ADT agent for mHSPC, and although it is known to be effective, resistance to the treatment still occurs. In this study, to further investigate the mechanism involved in apalutamide resistance, we first constructed apalutamide-resistant PCa cell lines in AR-positive LNCaP and C4-2 cells (Fig. 1A). A CCK-8 assay was used to determine the IC_50_ of the LNCaP and C4-2 cells as 45.48 µM/L and 20.16 µM/L, respectively. The same experimental assay was employed to show that the apalutamide-resistant PCa cells (LNCaP-AP-R and C4-2-AP-R) significantly improved cell viability compared to the parental cells (LNCaP-P and C4-2-P) (Fig. 1B).

The apoptosis, proliferation, and migration rates of the apalutamide-resistant PCa cells were comprehensively evaluated via various assays to reveal their malignant biological behavior. First, flow cytometry confirmed that the apoptosis rate of the apalutamide-resistant PCa cells was significantly lower than that of the parental cells after 24 h of apalutamide treatment (Fig. 1C). Second, a transwell assay showed a significant improvement in the migration ability of the apalutamide-resistant PCa cells (Fig. 1D). Finally, plate clone formation showed that the proliferation ability of the apalutamide-resistant PCa cells was significantly higher than that of the parental cells (Fig. 1E).

This study found that apalutamide changed the ERS and intracellular Ca^2+^ levels. Fluo-4 AM immunofluorescence assay ascertained that apalutamide increased the release of Ca^2+^ in the endoplasmic reticula of the parental cells to activate ERS, while the level of Ca^2+^ in the apalutamide-resistant PCa cells was significantly decreased in line with the activation of protective autophagy (Fig. 1F). Similarly, western blotting showed a substantial reduction in the ERS-associated proteins (PERK, eIF2, and ATF3) in the apalutamide-resistant PCa cells compared to the parental cells (Fig. 1G).

The autophagic flux in the LNCaP-AP-R cells was measured using the mCherry-GFP-LC3B construct to confirm the relationship between apalutamide resistance and protective autophagy. The mCherry-GFP-LC3B dual fluorescent autophagy indicator system was used to label and track the changes in LC3 and autophagy flow. This technique enabled the observation of the transformation from autophagosomes to autophagolysosomes. As shown in Fig. 1H, apalutamide treatment for 24 h decreased the yellow puncta in the LNCaP-AP-R cells. This indicated that the level of autophagy flow was reduced significantly in the apalutamide-resistant LNCaP cells, blocking the autophagy process. In addition, it was confirmed via western blot that the expression levels of the autophagy related proteins Beclin1 and LC3 I/II in the LNCaP-AP-R and C4-2-AP-R cells were significantly increased (Fig. 1I), while the TEM assay confirmed that the number of autophagosomes in the LNCaP-AP-R cells was significantly higher than that in the LNCaP-P cells (Fig. 1J). Finally, CCK-8 showed that the autophagy levels of the LNCaP-AP-R and C4-2-AP-R cells could be synergically enhanced by the autophagy activator rapamycin (Rapa), or significantly reversed by the autophagy inhibitor 3-methyladenine (3-MA) (Fig. 1K). These results confirmed that the apalutamide-resistant PCa cells promoted survival and metastasis by activating the protective autophagy levels.

### CAPN2 is significantly under-expressed in apalutamide-resistant PCa cells and tissues

**Fig. 2:**
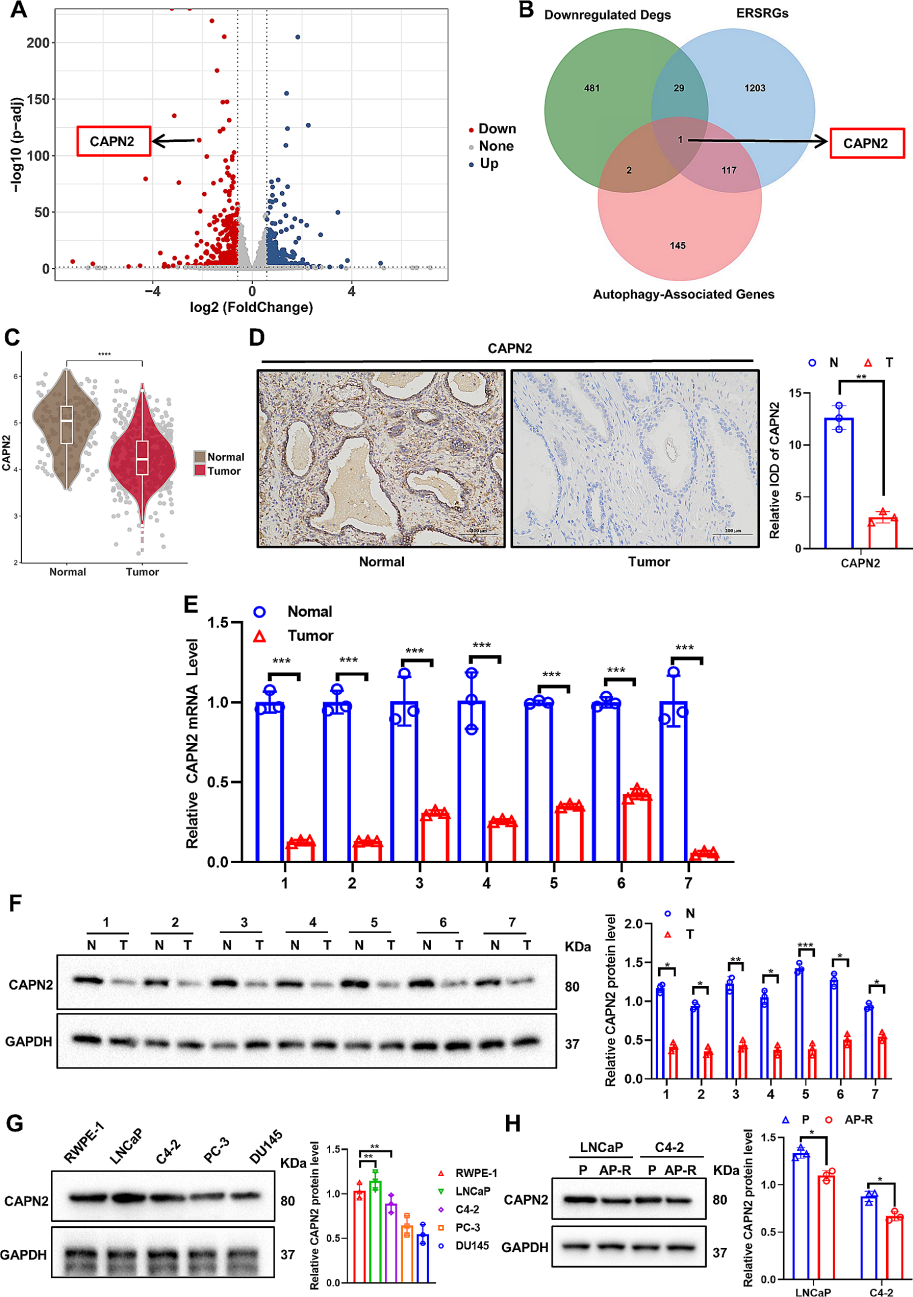
CAPN2 is significantly under-expressed in apalutamide-resistant PCa cells and tissues. (**A**) Volcano map showing genes with significant differences in transcriptomic sequencing of apalutamide-resistant PCa cells (blue dots represent up-regulated expression, red dots represent down-regulated expression, and gray dots represent no significant differences). (**B**) Revealing the common genes among the significantly downregulated gene set in apalutamide-resistant PCa cells, autophagy-associated gene set, and endoplasmic reticulum stress gene set (ERSRGs) through Veen diagram. (**C**) TCGA-PRAD data showed the expression level of CAPN2 in PCa and paracancer tissues. (**D**) The protein expression level of CAPN2 in PCa and adjacent tissues was detected by immunohistochemistry. (**E**) The mRNA expression of CAPN2 was detected by qRT-PCR in 7 pairs of PCa and adjacent tissues. (**F**) The protein expression level of CAPN2 was detected by western blot in 7 pairs PCa and adjacent tissues. (**G**) The expression level of CAPN2 in classical PCa cell lines (LNCaP, C4-2, PC-3 and DU145) and prostate epithelial cells (RWPE-1) was detected by western blot. (**H**) The expression level of CAPN2 in apalutamide-resistant PCa cells (LNCaP-AP-R and C4-2-AP-R) and the corresponding parental cells (LNCaP-P and C4-2-P)

Apalutamide-resistant PCa cell lines (LNCaP-AP-R and C4-2-AP-R) were constructed for RNA sequencing to further explore the mechanism involved in apalutamide resistance in PCa. The volcano map visualizing the results of the transcriptomic data analysis (Fig. 2A) shows genes with significant differences: the blue dots represent upregulated expression, the red dots denote downregulated expression, and the gray dots signify genes with no significant differences. The CAPN2 expression was significantly lower in the apalutamide-resistant PCa cells. This study primarily focused on CAPN2, as shown in the Venn diagram of the intersection between the downregulated DEGs, the ERS-related genes (ERSRGs), and the autophagy-associated genes (Fig. 2B). This study analyzed the Cancer Genome Atlas Prostate Adenocarcinoma (TCGA-PRAD) data to further verify the CAPN2 expression in the PCa tissues and apalutamide-resistant PCa cells. The results showed lower CAPN2 expression in the PCa than the paracancer tissues (Fig. 2C), while the immunohistochemistry (Fig. 2D), qRT-PCR (Fig. 2E), and western blotting (Fig. 2F) jointly confirmed substantially reduced CAPN2 protein and mRNA levels in the PCa tissues. Furthermore, the CAPN2 expression was determined in the classic PCa cell lines (LNCaP, C4-2, PC-3, and DU145) and prostate epithelial cells (RWPE-1). The results confirmed that the CAPN2 expression levels, especially in the LNCaP and C4-2 cells, were lower than in the RWPE-1 cells (Fig. 2G). Finally, we demonstrated that the CAPN2 expression was significantly lower in the LNCaP-AP-R and C4-2-AP-R cells than in the parental cells (Fig. 2H). In conclusion, the CAPN2 expression was significantly lower in the apalutamide-resistant PCa cells and tissues.

### Overexpression of CAPN2 significantly inhibits autophagy and metastasis of apalutamide-resistant PCa cells

**Fig. 3:**
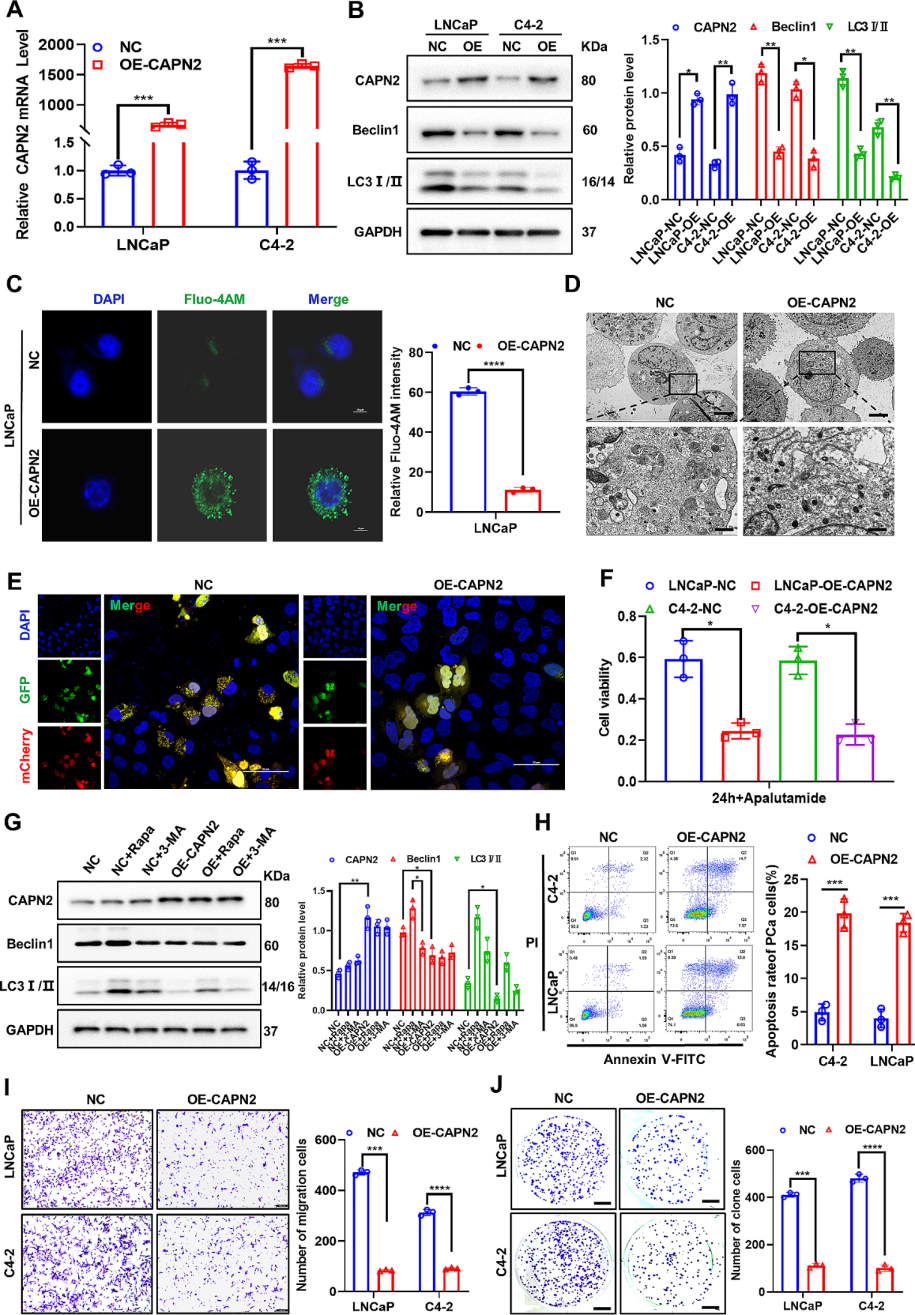
Overexpression of CAPN2 significantly inhibited autophagy and metastasis of apalutamide-resistant PCa cells. (**A**) The mRNA expression of CAPN2 after was detected by qRT-PCR after stable overexpression. (**B**) The protein expression of CAPN2 and autophagy related genes (Beclin1 and LC3 I/II) was detected by western blot after stable overexpression of CAPN2. (**C**) Intracellular calcium ions (Fluo-4AM) in stable overexpression of CAPN2 apalutamide-resistant PCa cells were confirmed by immunofluorescence assay. (**D**) The morphology, structure and number of autophagosomes in stable overexpression of CAPN2 apalutamide-resistant PCa cells was confirmed by TEM. (**E**) The level of autophagy flow after stable overexpression of CAPN2 was confirmed by immunofluorescence assay. (**F**) Apalutamide was added to stably overexpressed CAPN2 apalutamide-resistant PCa cells for 24 h and cell survival was measured by CCK-8 assay. (**G**) The autophagy related protein (Beclin1 and LC3I/II) level after the addition of an autophagy activator (Rapa) or an autophagy inhibitor (3-MA) to stably overexpressed CAPN2 apalutamide-resistant PCa cells, respectively, was confirmed by western blot. (**H**) The apoptosis rate of stably overexpressed CAPN2 apalutamide-resistant PCa cells was detected by flow cytometry assay. (**I**) The cell migration ability of stably overexpressed CAPN2 apalutamide-resistant PCa cells was confirmed by transwell experiment. (**J**) The proliferative ability of stably overexpressed CAPN2 apalutamide-resistant PCa cells was confirmed by plate clone formation assay

To further investigate the mechanism by which CAPN2 regulates apalutamide resistance in PCa, we constructed CAPN2 overexpressing plasmids and then stabilized their overexpression in LNCaP-AP-R and C4-2-AP-R cells. First, qRT-PCR was used to verify transfection efficiency, which showed that the CAPN2 mRNA levels increased 671- and 1646-fold in the LNCaP-AP-R and C4-2-AP-R cells, respectively (Fig. 3A). Western blotting subsequently revealed that the overexpression of CAPN2 in the LNCaP-AP-R and C4-2-AP-R cells significantly inhibited the expression of the autophagy-related proteins Beclin1 and LC3 I/II (Fig. 3B).

Immunofluorescence in our subsequent studies involving the effect of CAPN2 on autophagy demonstrated that the overexpression of CAPN2 significantly increased the intracellular Ca^2+^ levels (Fig. 3C). This implies that overexpression of CAPN2 can promote the intracellular Ca^2+^ and ER stress levels. TEM confirmed that the overexpression of CAPN2 significantly inhibited autophagosome formation in apalutamide-resistant PCa cells (Fig. 3D). Subsequent immunofluorescence showed that the overexpression of CAPN2 significantly inhibited autophagy flow in apalutamide-resistant PCa cells transfected with the mCherry-GFP-LC3B plasmid (Fig. 3E). Furthermore, CCK-8 indicated that the overexpression of CAPN2 in the apalutamide-resistant PCa cells significantly inhibited cell viability (Fig. 3F). The CAPN2 overexpression in cells supplemented with the autophagy activator Rapa and autophagy inhibitor 3-MA was examined to confirm its regulatory effect on autophagy. The results confirmed that the inhibition of autophagy via the overexpression of CAPN2 could be significantly synergically either promoted by 3-MA or reversed by Rapa (Fig. 3G). In addition, flow cytometry (Fig. 3H), transwell assaying (Fig. 3I), and plate cloning experiments (Fig. 3J) confirmed that the overexpression of CAPN2 significantly promoted apoptosis and inhibited its metastasis and proliferation. Therefore, these findings verified that the overexpression of CAPN2 reversed apalutamide resistance in PCa by inhibiting protective autophagy.

### CAPN2 activates protective autophagy by reducing FOXO1 degradation and promoting its nuclear translocation to transcriptionally regulate ATG5

**Fig. 4:**
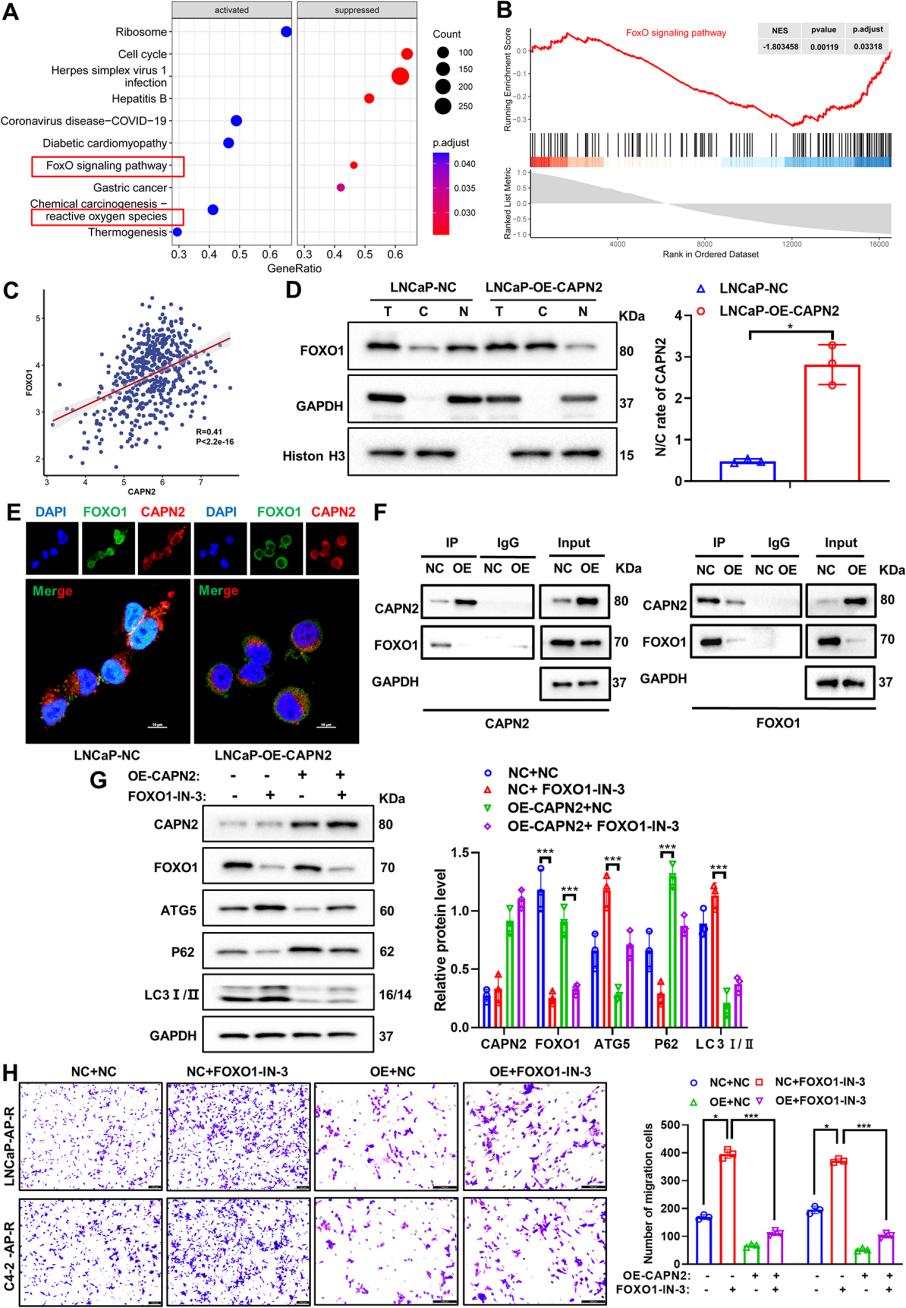
CAPN2 activates protective autophagy by reducing FOXO1 degradation and promoting its nuclear translocation to transcriptionally regulate ATG5. (**A**) KEGG pathway enrichment showed that CAPN2 was closely related to FOXO signaling pathway and reactive oxygen species pathway. (**B**) GSEA curve showed a significant positive correlation between CAPN2 and FOXO signaling pathway. (**C**) Spearman correlation analysis showed a significant positive correlation between CAPN2 and FOXO1. (**D**) Nucleoplasmic isolation assay showed that overexpression of CAPN2 in apalutamide-resistant PCa cells significantly inhibited nuclear translocation of FOXO1. (**E**) Immunofluorescence assay showed that overexpression of CAPN2 in apalutamide-resistant PCa cells significantly inhibited nuclear translocation of FOXO1. (**F**) CoIP experiment showed that overexpression of CAPN2 can significantly reduce its binding through degradation of FOXO1. (**G**) Under the premise of overexpression of CAPN2 in apalutamide-resistant PCa cells, FOXO1 inhibitor (FOXO1-IN-3) was added to detect the expression level of autophagy related proteins (Beclin1, P62 and LC3I/II) by western blot assay. (**H**) Under the premise of overexpression of CAPN2 in apalutamide-resistant PCa cells, FOXO1 inhibitor (FOXO1-IN-3) was added to detect the migration ability by tanswell experiment

In our further exploration of the target and mechanism of CAPN2-mediated protective autophagy, we first confirmed the close relationship between apalutamide resistance differential genes and the FOXO pathway through KEGG pathway enrichment (Fig. 4A). Next, the single gene set enrichment analysis (GSEA) curve was employed to verify the significant positive correlation between the FOXO pathway and CAPN2 (Fig. 4B). Similarly, spearman correlation analysis revealed a significant positive correlation between FOXO1 and CAPN2 gene expression, with a correlation coefficient of *R* = 0.41 (Fig. 4C). Nucleoplasmic isolation (Fig. 4D) and immunofluorescence experiments (Fig. 4E) further revealed the mechanism behind the CAPN2 regulation of FOXO1 and confirmed that CAPN2 inhibited the function of the transcription factor in the nucleus by binding with and degrading FOXO1. Next, this study showed that CAPN2 bound to FOXO1 and mediated its degradation and nuclear translocation. This was established via a CoIP experiment, indicating that CAPN2 overexpression significantly reduced the binding process via FOXO1 degradation (Fig. 4F). In addition, the FOXO1 inhibitor FOXO1-IN-3 were added to apalutamide-resistant PCa cells exhibiting CAPN2 overexpression to confirm the role of FOXO1 in autophagy. The results showed that CAPN2 overexpression significantly inhibited autophagy, while the FOXO1 inhibitors (FOXO1-IN-3) synergically restricted this biological effect (Fig. 4G). The corresponding experimental results were confirmed via the transwell assay. Overexpression of CAPN2 and inhibition of FOXO1 significantly restricted the migration ability of the apalutamide-resistant PCa cells (Fig. 4H). These results verified the ability of CAPN2 to reverse apalutamide resistance in PCa cells by degrading FOXO1 and inhibiting its nuclear translocation to weaken protective autophagy.

### FOXO1 enhances autophagy in apalutamide-resistant PCa cells by promoting ATG5 transcription

**Fig. 5:**
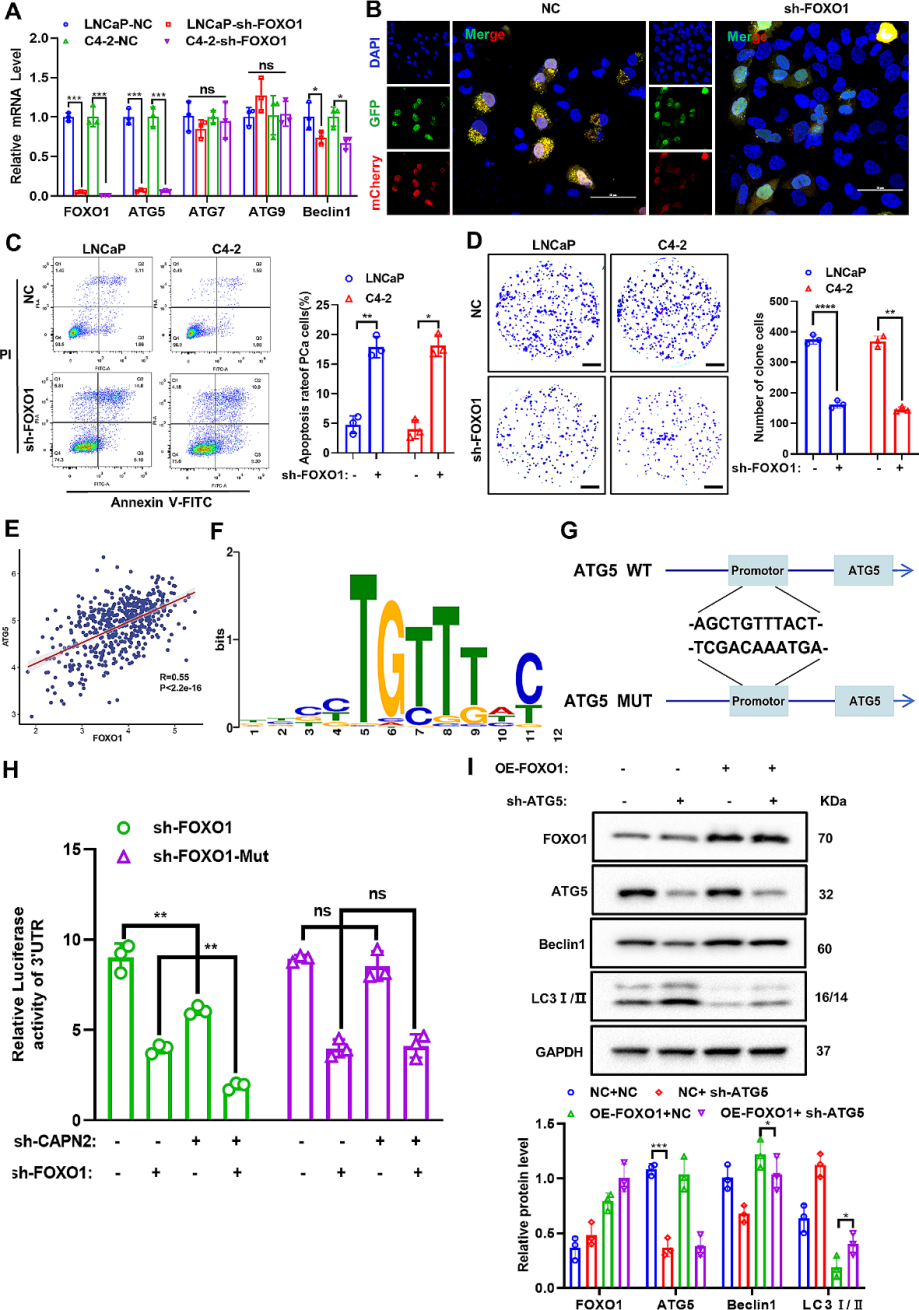
FOXO1 enhances autophagy in apalutamide-resistant PCa cells by promoting ATG5 transcription. (**A**) qRT-PCR was used to detect the efficiency of FOXO1 knockdown and its effect on the expression of autophagy related genes (ATG5, ATG7, ATG9 and Beclin1). (**B**) The level of autophagy flow after stable knockdown of FOXO1 was confirmed by immunofluorescence assay. (**C**) The apoptosis rate after stable knockdown of FOXO1 was detected by flow cytometry assay. (**D**) The cell migration ability of stably knockdown of FOXO1 apalutamide-resistant PCa cells was confirmed by tanswell experiment. (**E**) Gene correlation analysis showed a significant positive correlation between FOXO1 and ATG5. (**F**) JASPAR database predicted the binding site sequence of FOXO1 and ATG5. (**G**) The binding site of FOXO1 and ATG5 and the sequence of point mutations. (**H**) Double fluorescence reporter gene experiment revealed ATF3 transcriptional regulation of ATG5. (**I**) The effects of ATG5 knocking down on autophagy pathway proteins (ATG5, Beclin1 and LC3B) under the premise of overexpression of FOXO1 were detected by western blot assay

Next, a more thorough investigation was conducted of the molecular mechanism by which FOXO1 regulated autophagy. First, the FOXO1 was knocked down in the apalutamide-resistant PCa cells (LNCaP-AP-R and C4-2-AP-R), followed by qRT-PCR, to confirmed that the preliminary FOXO1 knockdown significantly reduced the transcription level of autophagy-related gene ATG5 (Fig. 5A). Further immunofluorescence experiments indicated that FOXO1 knockdown substantially inhibited the level of autophagy flow in apalutamide-resistant PCa cells (Fig. 5B). Subsequently, we further ascertained via flow cytometry that knocking down FOXO1 can significantly improve the apoptosis level in apalutamide-resistant PCa cells (Fig. 5C) and restricted their migration ability (Fig. 5D).

Based on the results showing that FOXO1 knockdown significantly inhibited ATG5 mRNA expression, it was reasonably assumed that FOXO1 likely regulated the transcription level of ATG5 as a transcription factor. Accordingly, database prediction indicated a significant positive correlation (*R* = 0.55) between the FOXO1 and ATG5 gene expression levels (Fig. 5E). The JASPAR database was used to further predict the binding site of FOXO1 and ATG5 promoters (Fig. 5F). Additionally, to prove the efficiency and role of the binding site, a mutation sequence of the binding site was designed, and a model diagram was drawn (Fig. 5G). Next, a double-fluorescent reporter gene assay was employed to confirm the ability of FOXO1 to promote the expression of ATG5 mRNA through transcriptional regulation, which enhanced the level of protective autophagy (Fig. 5H). Finally, rescue western blotting verified that the combined reduction of FOXO1 and CAPN2 in apalutamide-resistant PCa cells effectively suppressed the expression and activity of the autophagy pathway proteins. All these findings confirmed that FOXO1 enhanced the level of protective autophagy by promoting the expression of ATG5 transcription levels.

### ATF3 inhibits autophagy activation through the transcriptional regulation of CAPN2

**Fig. 6:**
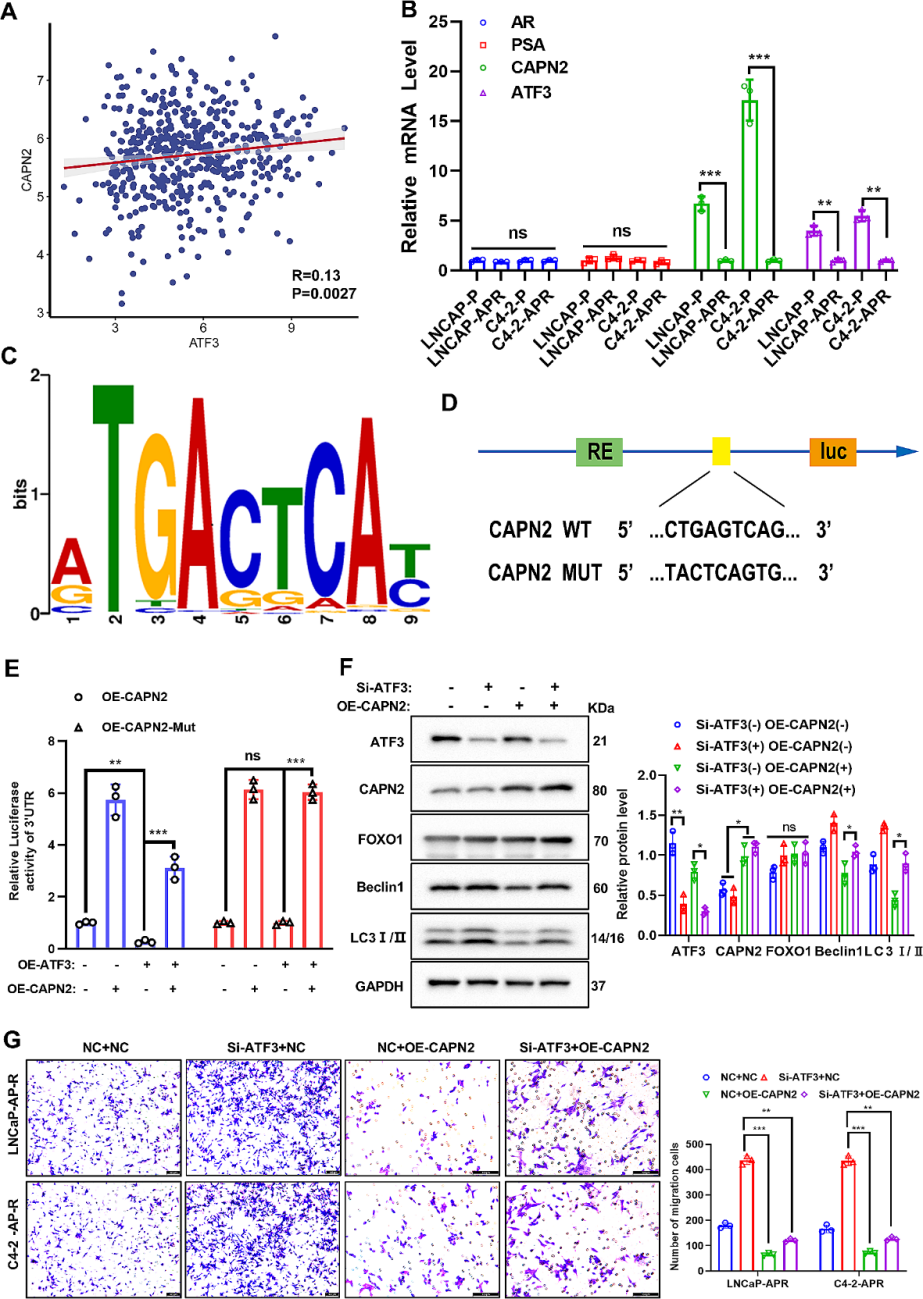
ATF3 inhibits autophagy activation through transcriptional regulation of CAPN2. (**A**) Gene correlation analysis showed a significant positive correlation between ATF3 and CAPN2. (**B**) The levels of AR, PSA, ATF3 and CAPN2 mRNA in apalutamide-resistant PCa cells and parental cells were detected by qRT- PCR. (**C**) JASPAR database predicted the binding site sequence of ATF3 and CAPN2. (**D**) The binding site of ATF3 and CAPN2 and the sequence of point mutations. (**E**) Double fluorescence reporter gene experiment revealed ATF3 transcriptional regulation of CAPN2. (**F**) Knocking down ATF3 in apalutamide-resistant PCa cells overexpressing CAPN2 revealed the mechanism of ATF3 regulation of CAPN2 and autophagy pathway proteins (Beclin1 and LC3I/II) through western blot experiments. (**G**) Knocking down ATF3 in apalutamide-resistant PCa cells overexpressing CAPN2 revealed the regulatory mechanism of ATF3 and CAPN2 on cell migration through transwell assay

Prior research has established that the regulation of CAPN2, a proteolytic enzyme, is influenced by intracellular Ca^2+^ levels in apalutamide-resistant PCa cells, elucidating the mechanism behind its low expression. This study demonstrated that apalutamide effectively stimulated the release of calcium and ERS in the parental PCa cells, while the opposite was observed in the apalutamide-resistant PCa cells (Fig. 1). Based on the significant reduction of transcription factor ATF3 in apalutamide-resistant PCa cells, this work predicted and subsequently confirmed the correlation between ATF3 and CAPN2 through correlation analysis (Fig. 6A). Furthermore, qRT-PCR confirmed a simultaneous decline of ATF3 and CAPN2 mRNA in the apalutamide-resistant PCa cells (Fig. 6B). Subsequently, the JASPAR database was employed to predict the potential of ATF3 to function as a transcription factor for CAPN2, as evidenced by the ATF3/CAPN2 binding site sequence (Fig. 6C). Figure 6D shows the ATF3/CAPN2 binding pattern and the binding site mutation sequence. Additionally, this study demonstrated that the overexpression of ATF3 significantly stimulated CAPN2 transcription through dual fluorescent reporter genes, while the mutation of their binding site significantly reversed this effect (Fig. 6E). Western blotting (Fig. 6F) and transwell experiments (Fig. 6G) demonstrated that simultaneous the knockdown ATF3 in the CAPN2-overexpressed apalutamide-resistant PCa cells reversed the inhibitory effect of CAPN2 overexpression on protective autophagy and migration. In summary, this study confirmed that ATF3 activated autophagy via transcriptional regulation of CAPN2.

### In vitro overexpression of CAPN2 significantly inhibits the proliferation of prostate cancer

**Fig. 7:**
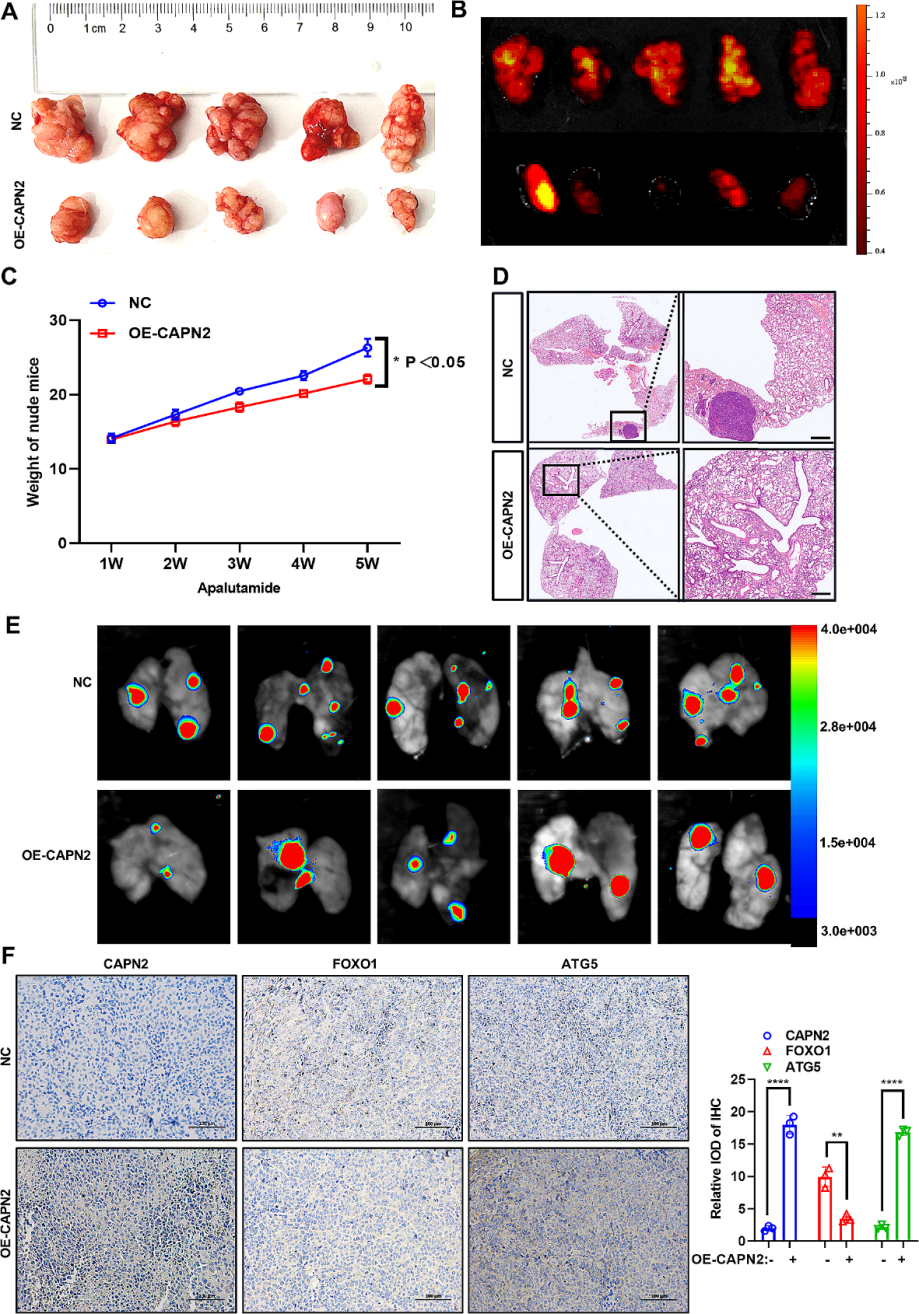
Overexpression of CAPN2 significantly inhibited the proliferation of prostate cancer in vitro. (**A**) The effect of CAPN2 overexpression on subcutaneous tumor proliferation in nude mice was revealed in vitro. (**B**) Bioluminescence in vivo imaging showed that overexpression of CAPN2 significantly decreased the fluorescence intensity of subcutaneous tumors (*n* = 5 per group). (**C**) The growth curve revealed the effect of CAPN2 overexpression on body weight gain in nude mice. (**D**) H&E staining indicated that overexpression of CAPN2 led to decreased number of lung metastatic colonies (*n* = 5 per group). (**E**) Bioluminescence in vivo imaging showed that overexpression of CAPN2 significantly decreased thed the number of pulmonary metastasis focuses (*n* = 5 per group). (**F**) The effect of overexpression of CAPN2 on the expression levels of CAPN2, FOXO1 and ATG5 in subcutaneous tumors of nude mice was detected by immunohistochemistry

**Fig. 8:**
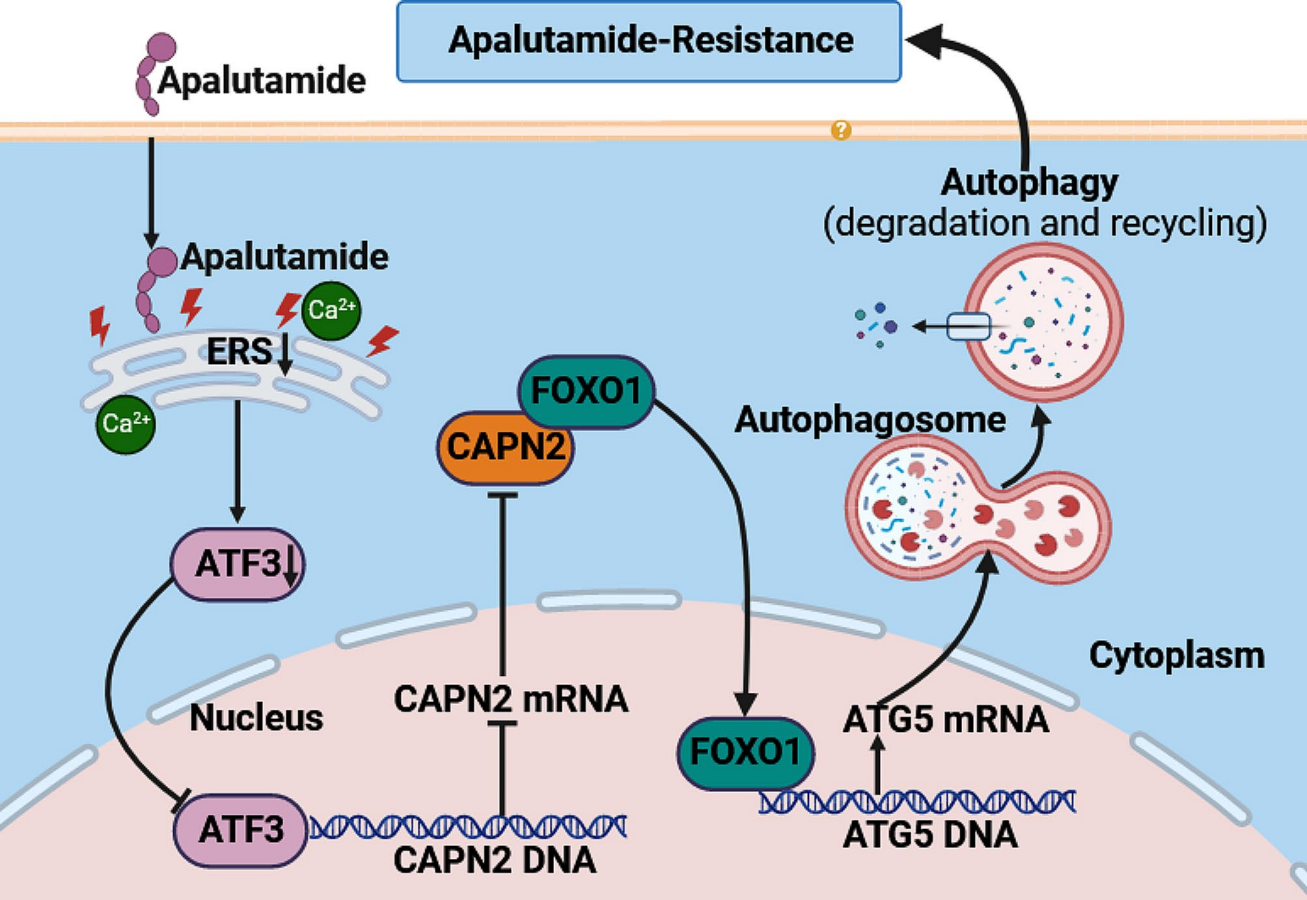
Mechanism model of CAPN2-mediated protective autophagy promoting apalutamide resistance in PCa

The specific role of CAPN2 in apalutamide resistance and protective autophagy activation was confirmed via in vitro experimentation. As shown in Fig. 7A, the stable overexpression of CAPN2 in apalutamide-resistant PCa cells significantly inhibited the proliferation of subcutaneous tumors, while in vivo bioluminescent imaging (BLI) revealed that it substantially decreased the fluorescence intensity of these tumors (Fig. 7B). Next, examination of the growth curves of nude mice indicated that the average body weights of those in the CAPN2 overexpression group were significantly lower than in the control group (Fig. 7C). Furthermore, H&E staining indicated that the overexpression of CAPN2 decreased the number of lung metastatic colonies (Fig. 7D). In vivo, the BLI showed that overexpression of CAPN2 significantly decreased the number of pulmonary metastatic focuses (Fig. 7E), while immunohistochemistry confirmed that the expression levels of CAPN2 and ATG5 in the overexpressed CAPN2 group were significantly higher than in the control group, with the FOXO1 expression levels displaying the opposite response (Fig. 7F). Finally, this study mapped the mechanical model of how CAPN2 promoted apalutamide resistance in PCa cells by activating protective autophagy (Fig. 8). In summary, the in vitro experiments further confirmed that the overexpression of CAPN2 reversed the proliferation and tumor-forming characteristics of apalutamide-resistant PCa cells, demonstrating its potential as a therapeutic target to enhance apalutamide treatment sensitivity.

## Discussion

Autophagy is a vital cellular process that protects cells and organisms from stress, and is usually induced as a pro-survival response to multiple cytotoxic therapies [13]. During the late stage in the progression of cancer, autophagy functions as a protective cellular defense mechanism, sustaining tumor metabolism, survival and development, and creating resistance to therapeutic agents [14]. PCa cells use autophagy as a survival mechanism in response to apalutamide treatment. This induction of autophagy is associated with increased expressions of Beclin1 and ATG5 proteins, and with the autophagy-related conversion of the microtubule-associated protein light chain 3 (LC3I to LC3II), the main regulators of autophagy in PCa [15]. Classic studies have indicated that at least two mechanisms underlie ADT-stimulated autophagy: one in which ADT induces local vasculature degeneration and hypoxia; and another in which, upon energy deficiency caused by ADT, AMP-activated protein kinase (AMPK) activation leads to mTOR pathway suppression and the promotion of autophagy [16].

In this study, we found that CAPN2 first activated protective autophagy and, subsequently, promoted tolerance to apalutamide in mHSPC cells. As a Ca^2+^-dependent cysteine protease, the activity and function of CAPN2 is dependent on the concentration of intracellular Ca^2+^. Since ER holds the major pool of Ca^2+^, ERS should promote Ca^2+^ efflux from ER and enhance CAPN2 activity [17]. The accumulation of Ca^2+^ into mitochondria depends on ER, the key intracellular Ca^2+^-storage organelle, and Ca^2+^ is released from the ER via the intracellular Ca^2+^-release channel, inositol 1,4,5-trisphosphate receptors (IP3Rs) and ryanodine receptors (RyRs). The inhibition of IP3R and consequent spontaneous Ca^2+^ signals compromise the mitochondrial bioenergetic, and lead to AMPK activation and ATG5 upregulation, resulting in excessive autophagy and cancer cell survival [18]. As a result, decreasing the ER Ca^2+^-store content by increasing IP3R1 levels was found to be a protective mechanism through which LNCaP cells can escape death upon androgen deprivation [19].

ATF3 is a member of the basic region leucine zipper (bZIP) family, and is an ERS response gene, as well as an adaptive response gene involved in DNA damage, cellular injury, and oxidative stress [20]. As a tumor suppressor gene, ATF3 has been proved to negatively regulate ATG5 genes by binding promoter regions, and might serve as a negative regulator of autophagy [21]. Here, we conclusively demonstrated that ATF3 promotes protective autophagy in apalutamide-resistant PCa cells, and that the silencing of ATF3 enhances CAPN2 expression by promoting its transcription. In esophageal cancer cells, ATF3 accumulation was found to induce protective autophagy and might serve as a survival signal [22], while in PCa cells, the AR degradation enhancer ASC-J9® could suppress cell proliferation and invasion by increasing ATF3 expression [23]. Therefore, a combination of ATF3-mediated CAPN2 inhibition and anti-androgen therapy may hold potential for the treatment of advanced PCa.

Forkhead box proteins (FOXOs) are a series of transcriptional factors that function as the regulator of genes involved in cell proliferation, differentiation and growth, among which FOXO1 is the most widely studied. The post-translational modification of FOXO1 vitally regulates its ability to activate certain genes related to apoptosis, DNA repair, and the defense against oxidative stress. Increased FOXO1 nuclear accumulation and transcriptional activity were observed upon histone deacetylase inhibitors (HDACIs) treatment, while the knocking-down of FOXO1 markedly reduced HDACIs-induced autophagy, indicating that FOXO1-mediated autophagy might be a novel therapeutic strategy for cancer therapy [24]. Furthermore, as a downstream effector of the PTEN tumor suppressor, FOXO1 has previously been found to block the transcriptional activity of either full-length AR or the active splice variants of AR in PCa, thus representing a pivotal molecular target for PCa therapy [25]. In this present study, CAPN2 was shown to activate protective autophagy by reducing FOXO1 degradation and promoting its nuclear translocation, thus revealing a novel function of FOXO1 in CAPN2-mediated autophagy in PCa cells. However, the detailed mechanisms underlying the way in which FOXO1 may regulate protective autophagy pathways in the apalutamide-resistance of mHSPC warrant further investigation.

## Conclusion

In summary, this study comprehensively and intensively revealed that CAPN2 inhibits FOXO1 degradation and promotes its nuclear translocation to transcriptionally regulate ATG5, thereby activating protective autophagy and inducing apalutamide resistance in PCa cells. Moreover, this process is synergically promoted by ATF3 transcriptional regulation. The findings herein are expected to reveal a new mechanism of apalutamide resistance and, significantly, provide novel targets for its treatment.

### Electronic supplementary material

Below is the link to the electronic supplementary material.

Supplementary Material 1

Supplementary Material 2

## Data Availability

The datasets used and/or analyzed during the current study are available from the corresponding authors on reasonable request.

## Abbreviations

mHSPC: Metastatic hormone-sensitive prostate cancer
CRPC: Castration-resistant prostate cancer
PCa: Prostate Cancer
ADT: Androgen deprivation therapy
AR: Androgen receptor
AP-R: Apalutamide-resistance
CAPN2: Calpain 2
FOXO1: Forkhead Box O1
ATG5: Autophagy related gene 5
TEM: Transmission electron microscopy
ERS: Endoplasmic reticulum stress
ATF3: Activating Transcription Factor 3

## References

[1] Matsumura N, Fujita K, Nishimoto M, Minami T, Tahara H, Yoshimura K, Uemura H (2023). Current status and future perspectives of the managements of metastatic hormone-sensitive prostate cancer. World J Urol.

[2] Smith MR, Saad F, Chowdhury S, Oudard S, Hadaschik BA, Graff JN, Olmos D, Mainwaring PN, Lee JY, Uemura H, De Porre P, Smith AA, Brookman-May SD, Li S, Zhang K, Rooney B, Lopez-Gitlitz A (2021). Small, apalutamide and overall survival in prostate Cancer. Eur Urol.

[3] Chi KN, Chowdhury S, Bjartell A, Chung BH, Pereira de Santana Gomes AJ, Given R, Juarez A, Merseburger AS, Ozguroglu M, Uemura H, Ye D, Brookman-May S, Mundle SD, McCarthy SA, Larsen JS, Sun W, Bevans KB, Zhang K, Bandyopadhyay N (2021). Agarwal, Apalutamide in patients with metastatic castration-sensitive prostate Cancer: final survival analysis of the Randomized, Double-Blind, phase III TITAN Study. J Clin Oncol.

[4] Schmidt KT, Huitema ADR, Chau CH, Figg WD (2021). Resistance to second-generation androgen receptor antagonists in prostate cancer. Nat Rev Urol.

[5] Chen Y, Zhou Q, Hankey W, Fang X, Yuan F (2022). Second generation androgen receptor antagonists and challenges in prostate cancer treatment. Cell Death Dis.

[6] Debnath J, Gammoh N, Ryan KM (2023). Autophagy and autophagy-related pathways in cancer. Nat Rev Mol Cell Biol.

[7] Ashrafizadeh M, Paskeh MDA, Mirzaei S, Gholami MH, Zarrabi A, Hashemi F, Hushmandi K, Hashemi M, Nabavi N, Crea F, Ren J, Klionsky DJ, Kumar AP, Wang Y (2022). Targeting autophagy in prostate cancer: preclinical and clinical evidence for therapeutic response. J Exp Clin Cancer Res.

[8] Yu Y, Liu B, Li X, Lu D, Yang L, Chen L, Li Y, Cheng L, Lv F, Zhang P, Song Y, Xing Y (2022). ATF4/CEMIP/PKCalpha promotes anoikis resistance by enhancing protective autophagy in prostate cancer cells. Cell Death Dis.

[9] Fontana F, Moretti RM, Raimondi M, Marzagalli M, Beretta G, Procacci P, Sartori P, Montagnani Marelli M (2019). Limonta, delta-tocotrienol induces apoptosis, involving endoplasmic reticulum stress and autophagy, and paraptosis in prostate cancer cells. Cell Prolif.

[10] Li Y, He Z, Lv H, Chen W, Chen J (2020). Calpain-2 plays a pivotal role in the inhibitory effects of propofol against TNF-alpha-induced autophagy in mouse hippocampal neurons. J Cell Mol Med.

[11] Ma XL, Zhu KY, Chen YD, Tang WG, Xie SH, Zheng H, Tong Y, Wang YC, Ren N, Guo L, Lu RQ (2022). Identification of a novel calpain-2-SRC feed-back loop as necessity for beta-catenin accumulation and signaling activation in hepatocellular carcinoma. Oncogene.

[12] Liu T, Mendes DE, Berkman CE (2014). Prolonged androgen deprivation leads to overexpression of calpain 2: implications for prostate cancer progression. Int J Oncol.

[13] Kimmelman AC, White E (2017). Autophagy and Tumor Metabolism. Cell Metab.

[14] Li X, He S, Ma B (2020). Autophagy and autophagy-related proteins in cancer. Mol Cancer.

[15] Eberli D, Kranzbuhler B, Prause L, Baumgartner V, Preda S, Sousa R, Lehner F, Salemi S (2022). Apalutamide and autophagy inhibition in a xenograft mouse model of human prostate cancer. J Cancer Res Clin Oncol.

[16] Ziparo E, Petrungaro S, Marini ES, Starace D, Conti S, Facchiano A, Filippini A, Giampietri C (2013). Autophagy in prostate cancer and androgen suppression therapy. Int J Mol Sci.

[17] Xie RJ, Hu XX, Zheng L, Cai S, Chen YS, Yang Y, Yang T, Han B, Yang Q (2020). Calpain-2 activity promotes aberrant endoplasmic reticulum stress-related apoptosis in hepatocytes. World J Gastroenterol.

[18] Ivanova H, Kerkhofs M, La Rovere RM, Bultynck G (2017). Endoplasmic reticulum-mitochondrial ca(2+) fluxes Underlying Cancer Cell Survival. Front Oncol.

[19] Boutin B, Tajeddine N, Monaco G, Molgo J, Vertommen D, Rider M, Parys JB, Bultynck G, Gailly P (2015). Endoplasmic reticulum ca(2+) content decrease by PKA-dependent hyperphosphorylation of type 1 IP3 receptor contributes to prostate cancer cell resistance to androgen deprivation. Cell Calcium.

[20] Chen M, Liu Y, Yang Y, Qiu Y, Wang Z, Li X, Zhang W (2022). Emerging roles of activating transcription factor (ATF) family members in tumourigenesis and immunity: implications in cancer immunotherapy. Genes Dis.

[21] Sood V, Sharma KB, Gupta V, Saha D, Dhapola P, Sharma M, Sen U, Kitajima S, Chowdhury S, Kalia M, Vrati S (2017). ATF3 negatively regulates cellular antiviral signaling and autophagy in the absence of type I interferons. Sci Rep.

[22] Liang Y, Jiang Y, Jin X, Chen P, Heng Y, Cai L, Zhang W, Li L, Jia L (2020). Neddylation inhibition activates the protective autophagy through NF-kappaB-catalase-ATF3 Axis in human esophageal cancer cells. Cell Commun Signal.

[23] Tian H, Chou FJ, Tian J, Zhang Y, You B, Huang CP, Yeh S, Niu Y, Chang C (2021). ASC-J9(R) suppresses prostate cancer cell proliferation and invasion via altering the ATF3-PTK2 signaling. J Exp Clin Cancer Res.

[24] Zhang J, Ng S, Wang J, Zhou J, Tan SH, Yang N, Lin Q, Xia D, Shen HM (2015). Histone deacetylase inhibitors induce autophagy through FOXO1-dependent pathways. Autophagy.

[25] Zhao Y, Tindall DJ, Huang H (2014). Modulation of androgen receptor by FOXA1 and FOXO1 factors in prostate cancer. Int J Biol Sci.

