# Supplementary material for: CAPN2 promotes apalutamide resistance in metastatic hormone-sensitive prostate cancer by activating protective autophagy
Source: J Transl Med. 2024 Jun 6;22:538. doi: 10.1186/s12967-024-05335-z (PMC11155045; doi:10.1186/s12967-024-05335-z)
Supplement: Supplementary file 1 — Supplementary Material 1 [file 12967_2024_5335_MOESM1_ESM.docx]

**Supplementary Table 1. The PCR primer sequences of the gene in this study**

| **Gene name** | **Primer sequences** |
| --- | --- |
| AR | 5’-CGTCCTCTCCGGAACTGAT-3’  3’-TCCTGCTTCCAAAGTCCATT-5’ |
| PSA | 5’-CGTGACGTGGATTGGTGC-3’  3’-GCCGCAGACTGCCCTG-5’ |
| ATF3 | 5’-CGCTGGAATCAGTCACTGTCAG-3’  3’-CTTGTTTCGGCACTTTGCAGCTG-5’ |
| CAPN2 | 5’-GTTCTGGCAATACGGCGAGT-3’  3’-CTTCGGCTGAATGCACAAAGA-5’ |
| FOXO1 | 5’-GGGTTAGTGAGCAGGTTACAC-3’  3’-TCCAATGGCACAGTCCTTATC-5’ |
| GAPDH | 5’-AATGAAGGGGTCATTGATGG-3’  3’-AAGGTGAAGGTCGGAGTCAA-5’ |

**Supplementary Table 2. Primary and secondary antibodies in this study**

| **Gene Names** | **Sources** | **Catalog number** | **Host** |
| --- | --- | --- | --- |
| AR Antibody | Proteintech | 22089-1-AP | Rabbit |
| KLK3/PSA Antibody | Proteintech | 10679-1-AP | Rabbit |
| GAPDH Antibody | Proteintech | 60004-1-Ig | Mouse |
| ATF3 Antibody | ABclonal | A13469 | Rabbit |
| CAPN2 Antibody | Proteintech | 11472-1-AP | Rabbit |
| FOXO1 Antibody | Proteintech | 18592-1-AP | Rabbit |
| PERK Antibody | Proteintech | 24390-1-AP | Rabbit |
| eIF2 Antibody | Proteintech | 10227-1-AP | Rabbit |
| Beclin1 Antibody | Proteintech | 11306-1-AP | Rabbit |
| LC3 Antibody | Proteintech | 14600-1-AP | Rabbit |
| ATG5 Antibody | Proteintech | 10181-2-AP | Rabbit |
| P62 Antibody | Proteintech | 18420-1-AP | Rabbit |
| HRP-conjugated Affinipure Goat Anti-Mouse IgG(H+L) | Proteintech | SA00001-1 | Mouse |
| HRP-conjugated Affinipure Goat Anti-Rabbit IgG(H+L) | Proteintech | SA00001-2 | Rabbit |
| CoraLite488-conjugated Goat Anti-Rabbit IgG(H+L) | Proteintech | SA00013-2 | Rabbit |
| CoraLite594-conjugated Goat Anti-Mouse IgG(H+L) | Proteintech | SA00013-3 | Mouse |

**Supplementary Table 3. Drugs and reagents used in this study**

| **Drug /Reagent** | **Source** | **Identifier / formulation** |
| --- | --- | --- |
| Annexin V-FITC/PI Apoptosis Detection Kit | Vazyme | A211-02 |
| Primary Antibody Dilution Buffer | Solarbio | A1810 |
| ChamQ Universal SYBR qPCR Master Mix | Vazyme | Q711-02 |
| HiScript III RT SuperMix for qPCR (+gDNA wiper) | Vazyme | R323-01 |
| Reactive Oxygen Species Assay Kit | Beyotime | S0033S |
| Dual Luciferase Reporter Assay Kit | Vazyme | DL101-01 |
| Fetal Bovine Serum (Charcoal Stripped) | Biological Industries | 04-201-1B |
| [Apalutamide](javascript:;) | MedChemExpress | HY-16060 |
| TrypLE™ Express Enzyme (1X), no phenol red | Gibco | 12604-013 |
| RPMI 1640 Medium | Gibco | C11875500BT |
| RPMI 1640 Medium, glutamine, no phenol red | Yuanpei Bio | L230KJ |
| Fetal Bovine Serum | Biological Industries | 04-001-1ACS |
| Penicillin-Streptomycin | Gibco | 15140122 |
| HiPure Total RNA Mini Kit | Magen | R4111-03 |
| Rapamycin | MedChemExpress | HY-10219 |
| 3-MA | MedChemExpress | HY-19312 |
| FOXO1-IN-3 | MedChemExpress | HY-153617 |
| Fluo-4-AM | MedChemExpress | [HY-101896](https://www.medchemexpress.cn/Fluo-4_AM.html) |

**Supplementary Table 4. The sequence of CAPN2 promoter**

| **Gene name** | **Promoter sequence** |
| --- | --- |
| **CAPN2** | **CAPN2 Promoter (-2000~-1)**  >5' Flanking sequence chromosome:GRCh38:1:223699593:223701592:1  GCCTCACCTCTGGCCTCTTCTGGCCCTTGACAAAGATTCTTTGCTTGACCAAACTTTAGTGAGCCTCCTGATCCTTTCCTAGGCCCATCTGTGCACTTCCTTGAAAAATTCAGTTTTAACGAGGACCCTGCTAAGTCAGTTTAGCTAGAACCCCTCAACACCTTGCTATCTGATCATCCCCAATATCTAACTGTGCTCCTCATCCTCCACCATCTAGGTGATGTCCAATGACCCTGTCCTGTCTTCAGCAAGACTCGGTTAGGGGGTTTAGCCAGAATCCCCCTTATTCTTTACGTTTACTCTTAGTAATTATCTGTCACTGACCCCCACTCTGCTTCTTGGCTATGCATTCCCACCTGGCCGTGCCATATTCCGAGTTGAGCCCAATCTCTATCCACCCCGCAAAACCCCATTGCAGTGGCCCCTATGCCTATTGCAACAGTCCTGAATAAAGCCTTCCTAATCATACCTCAACAAGTGCCACCGAATAATTTTTTCTTTTCTTTGACACCAATCTCACATTACTACCACCACCCTCCCCCAGGACCCTACTCCCAGAACACACTCAACCACCCCCATCCCTACATAGAATGCTCAGGGCGCCAAGGGGGCGTCCAGGGACTTCATGGTTTCTAGCACTTTGTAGTCCACCGAAATGCCCCCAGGAGTGGCAATCAAGTGGAGAGGCTGGGCTGTGTGTTGATGGCCAGTCTGGTCCCAAGTAGACCCCATAGAGATGTCCTGGGCCCTTCATCCCCCCTCCACACCAGCACCAGCACCCACAACTTAGGGACAGACCAGTCAGCCCCAGAGAGAGAGAGAAAGAGAGAGAGGGAGGAGGGAGCAACAAGAAATGCTTGCACAGCTCTGTTCTGCAACATTCTTATCTTTCTCCTAGAGGAATTCAAGCATTAAACCCTGGGGGAGGAATCTTCTGGTTCTCTTGTAAAGGAGGGTACACTCCACCCCACCCTAAACTCAGACATTTGAAGCAATCCTAGTGGATAGTAAATCTGGGCTCCTCAAAGTTCTCAATTCAGGGACTCCTTTAGCATGACACAGGGCCCAGGGAGCATTTCCCTCACCCTAAATCAATGATTCTGACATCAAAGATGCCAACAAGTTTTCCCTGGGAACGCTTTGTGCCTATGACCCTTCGTTCAACAAACCTTTACCAAGCACCCACCAGGTGCCAGGCACTATGCCAGGATCACAAAGGTGAACAAGAAAGACATGGTGTCTCATTCAGAAATGGATGGGCTAGCGGGGAAATGGACACTAAATATTTGTATCACCAGGTGATACGCGCTCTCACAGAAGTTACATAAAACGTGCCAAGATGACAGCTCTAATTATTCACTATTGTGCTTTTCAGTTTATATTCAAGTCCACATTGCAGAATACATGTTAGAGAGCACTTATGTCTTGACATGACTTGCACTTTTGATCAGTCCATAGACTGCTTGAGTGGAAAGAAATTCCTCTAAGACACTCCCTTCTGAGTATGAGTCAGAAAGCTGAGGTCTCCCCAGCATAGCCGGGTCAGGGGCAGGGTGACAGGGCCTCCCGACACACGCAGCATCCAAGCAGACTGAGCAGAGCCTGCAGAGCGGGCAGCAGGATGGGACCTCTCCCGAGAGTCTGGAAGCCCCAGCTGGCTCCGGCCCTCTCTAGGGCTGCGGGACCCTCCCTTCTCTGGCCTCAGCTTCTTAGCTGCCTTGGTTAGACTGGACAGGAGGAGACAGCTCTGCCCTCCTTTCCACCTCTGAGCTTATGCTCTTGTGAGTCTCATTCTTGCCATGAAGAATTGAAGAATCTTTGTGCTCTGAGTCAGAAGCTATGTGGCCGCTTTGCTAAAATGTGACTCAAACACAGCCTCAGCCCTGAGGCCCCTTTCATTATATTCCCTTATTTGGTCCCAGGCAGCATTTCCATATTCTCTCTGGGGAGCTGGTGGGCAGAGCCTTTCTGTGGGCCAAGGAGGAGCTCAGGTGCCAGAC |

**Supplementary Table 5. ATF3 binding site sequence and mutation sequence of CAPN2**

| **Gene name** | **Binding site sequence** | **Mutation sequence** |
| --- | --- | --- |
| CAPN2 | CTGAGTCAG | TACTCAGTG |

**Supplementary Table 6. FOXO1 binding site sequence and mutation sequence of ATG5**

| **Gene name** | **Promoter sequence** |
| --- | --- |
| **ATG5** | **ATG5 Promoter (-2000~-1)**  >5' Flanking sequence chromosome: GRCh38:6:106325792: 106327791: -1  CAGGAAGTTGAATTTTAAGTCAAATCAAGTAATCACCTTTCACCTATTGGGTTTCTTCATACATAGTTTCCCCCAAAACAATTTTTTTTTTTTTTTGAGACAGGGTCTCTCTCTGTTACCTAGACTGCAGTGATACAATCATAGCTCACTGCAACCTTGACCTCCTGGGCTCATGTAATCCTCCTTCCTCAACCACTGGAGTAGCTGGGACTGTAGGCGCATGCCACCATGCCCTCCTAATTTTTGTGTTTTTTGTAGAGATGTGGTCTTGCCATGTTGTGCAGGCTGGTCTTGAACTCCTGGGCTCAAGCTATCCTCTTACCTGGGCCTCCCAAAGTGCTGGGATTACAGGCATGAGCCACTGTGCCTGGTCCCAACATTTTGTTAGGAAATAATTTTGAACACACAGCAAAGCTGAACAAATTTTACAGTGAATACCCATTTACAATGAACCTAGATTCTACCATCTAGATTCTACCATTATATTTTACCATTATATTCTACCATTATATTTTACTATATTTGGTTAATCACATATTTATCCATCTATGCGCCCCTAAATCCATCTTATTTTTTAGATACATTTTAAAGGAAATTTTATATACTTTTGTCTGACACTGGGAAGGCAATGCACCTTAATCCCACAAGCTTTCTTACAGAGGCTAATAGCTGTTTACTGGGGACACTGTAGTGAGGATTTCTGCATTGAGTAGGTGAAGAGGACCATAACTGTGGCTTTCAAACTTTTCTGACTGCTACCCTTACTAAGAAATGTATTTCACATTCCTATCCAGTGTACACCACATACATACTGAAGTAAAAATTTTGGGACACAATATTTACCCTTTACATGTGTGAAGCATGCTGATATTTTCTATTTTATTTCATTTAAAAAGATGCTGGGCATGCTTCCCTAACTTGATTTAGATACCCACTAATGTACTGTAATTTACCATTTGAAAATCATTGTACTTAGTCCAACTCCAAGAAGATTTTTCTTTTTAAAAGAATCCTGTGGCTACTACTAGAATTTTCCTTATGTTCCCAATTATCAAGAACCTGTTTTGAGTCTCAGCACAGTACTTACTTAACATGCATGGCGTTAAGGCACGTGTAATTAAGAGAAAAACCTTAAATGCCTGCTAAGACTTCTGGATAGAGCCAATTCAAAGATTCTTTATTAGTAAGTTTACCAATTATTACTATTTTTTTTTTGAGACGGAGTCTCGCTCTTTCGCCCAGGCGGGACTGCGGTGGCCCTATCTTGGCTCACTGCAAGCTCCACCTCCCGGGTTCACGCCATTCTCCTGCCTCAGACTCTCCAGTAGCTGGGACTACAGAAGCCCGCCACCGCGCCCGGCTAATTTTTTTTGTATTTTTAGTAGAGACGGGGTTTCACCGTGTTAGCCATGATGGTCTCGATCTCCTGACCTCGTGATCCGCCCGCCTCGGCCTCCCAAGGTGCTGGGATTACAGGCGTGAGCCACCGCGCCCGGCCCCAATTATTTTTACAAAGAGTAAGATAAAGATATGTACTCTTGGATGGGTGGGAGGGTTCAGTGCTCATATAGTCTTCTGTTAAATTAGGTTATTAATGCCTGCGGTGGTTCCAACAAAGTA  GAGAAGAAGATCAAATAAGAAAATGGATGGGAAAGTACTTTGAAAACTAATGTTTTATTAATACTGTTGTGTGATGCCAGGAAGAAAGGGATGTTGCGATCAAGCTCTAGGGAGGCAGTGTTTCAGTATTTTAAAAACTGTTTTTGTGGAACTGTTAAGTTAACCTCCCCAAGCCCCAATAACTACGCTTTGACGCAAGATTCTCAGAAGAGGGTAGAAGCTATGAACTTGGAGAGGTTTGGTCGCGAGTTCAAGCGTACTGCCCCGTTGGCGCATGCTCAAGAGCGACTCGGGCCTCCTTGCTGGGTTAGGCAGAACACGGAGGGCGGAGTTCGGACCACGTGGGGTCTCGTGACGTCATCTCCGGGCGCCGAGGGTGA |

**Supplementary Table 7. FOXO1 binding site sequence and mutation sequence of ATG5**

| **Gene name** | **Binding site sequence** | **Mutation sequence** |
| --- | --- | --- |
| ATG5 | AGCTGTTTACT | TCGACAAATGA |
