# Supplementary material for: CAPN2 promotes apalutamide resistance in metastatic hormone-sensitive prostate cancer by activating protective autophagy
Source: J Transl Med. 2024 Jun 6;22:538. doi: 10.1186/s12967-024-05335-z (PMC11155045; doi:10.1186/s12967-024-05335-z)

Figure.1G PERK


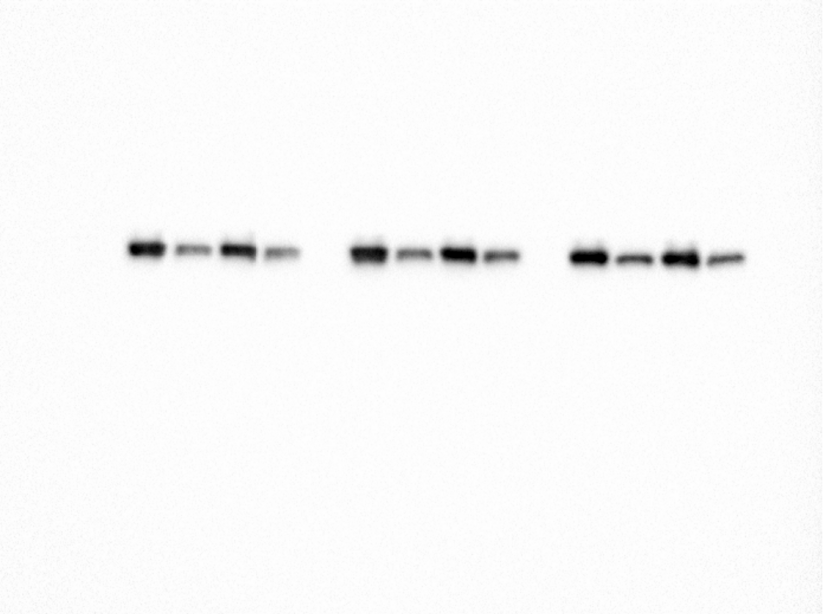


Figure.1G eIF2


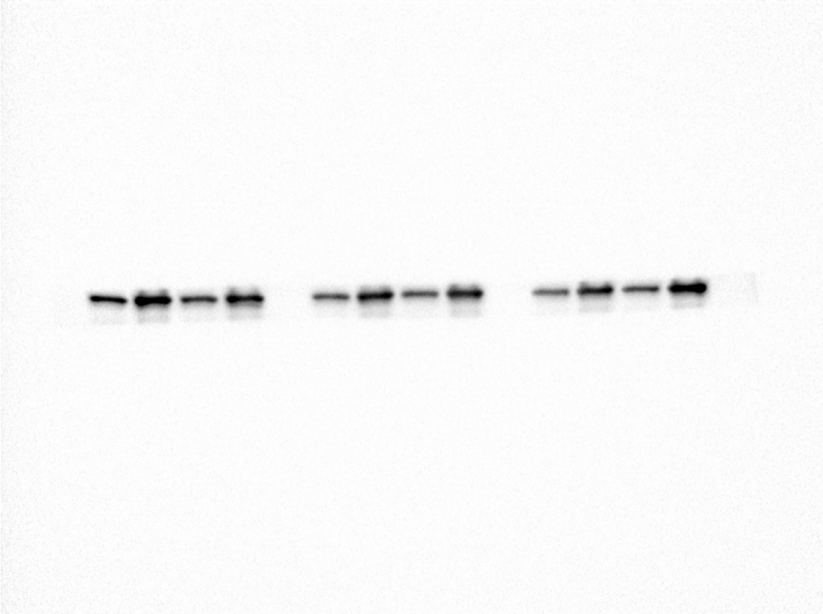


Figure.1G ATF3


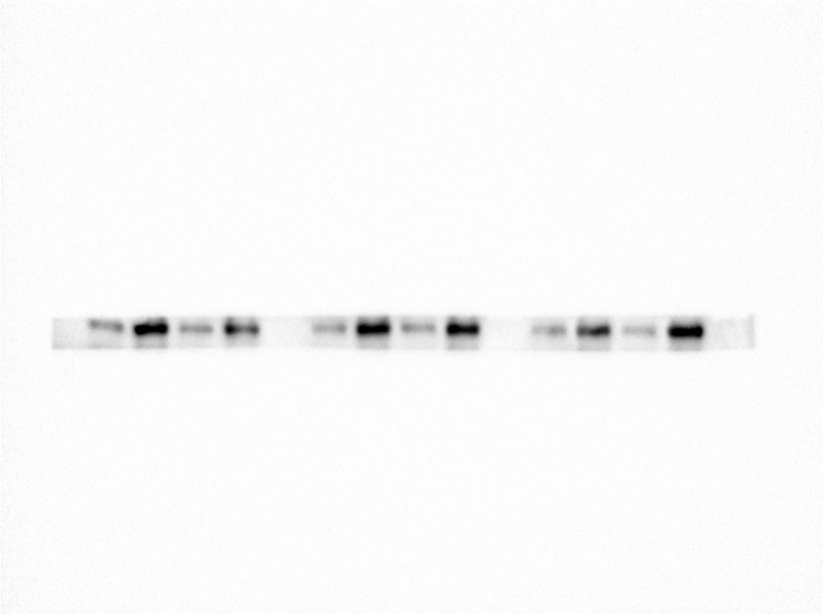


Figure.1G GAPDH


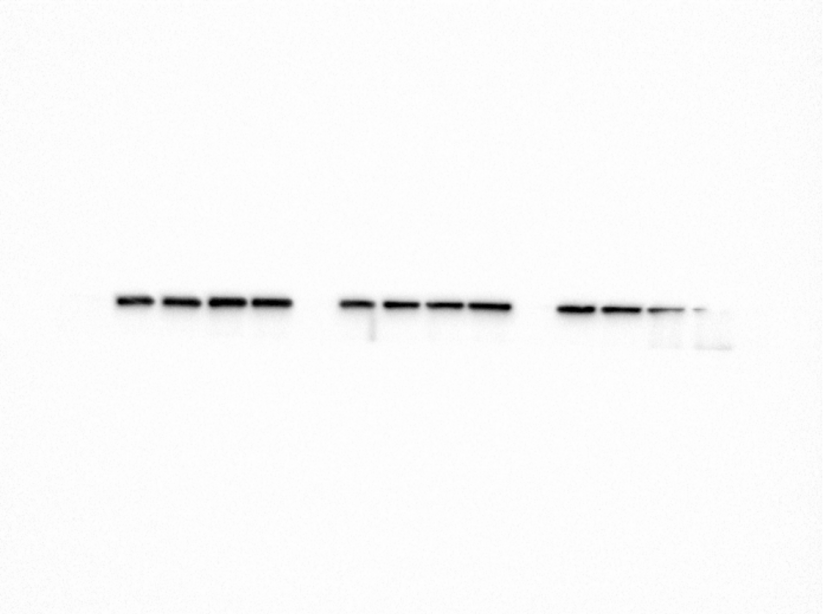


Figure.1I AR


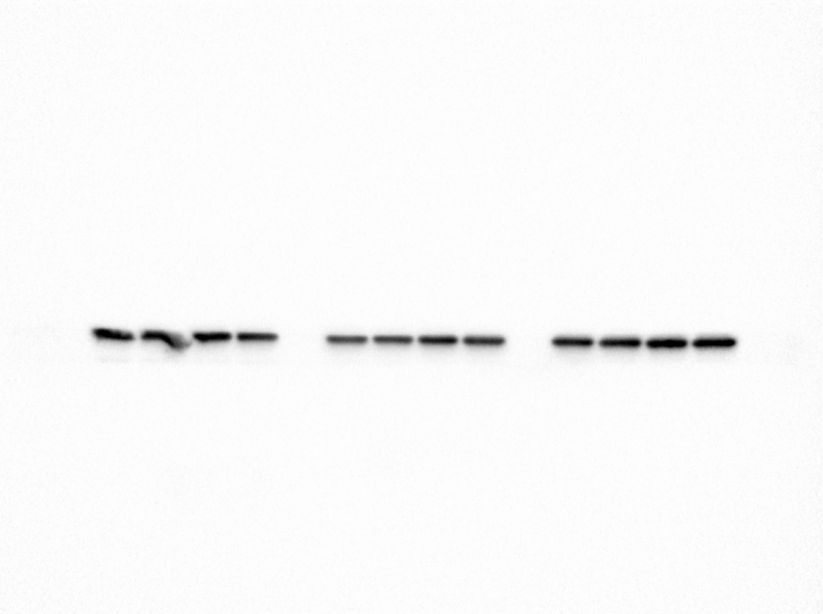


Figure.1I PSA


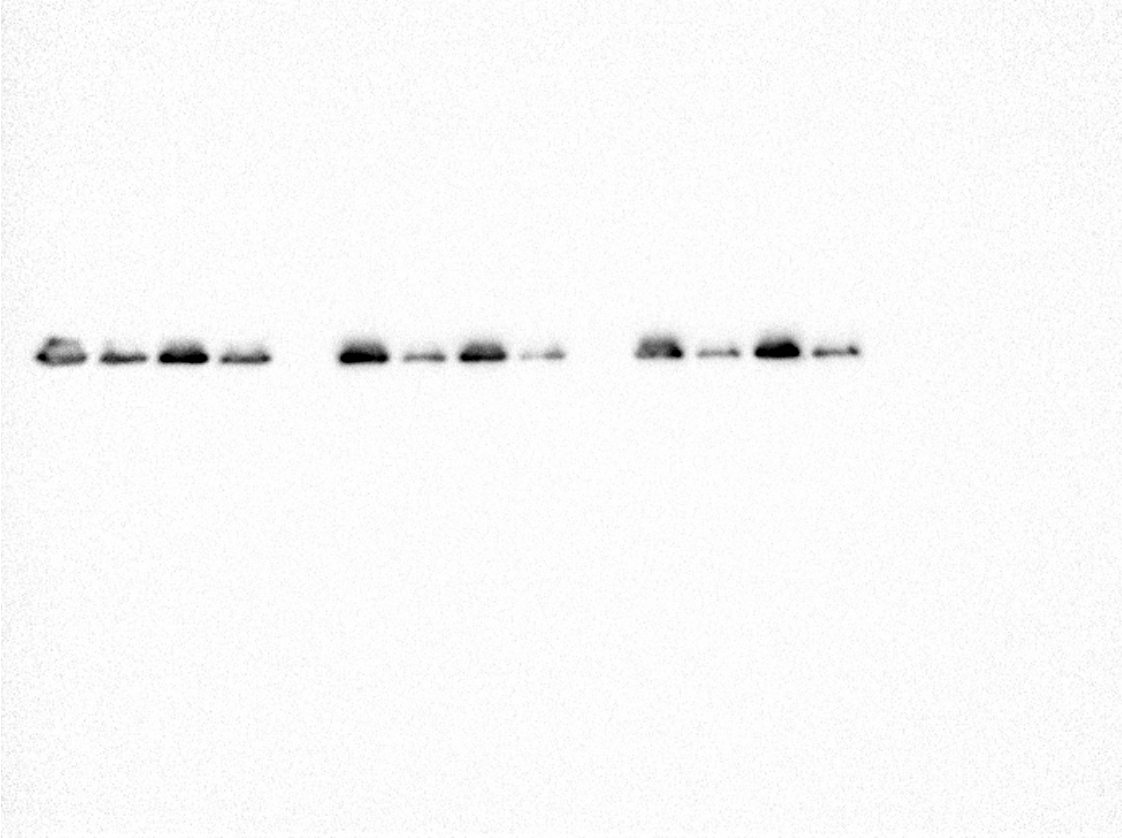


Figure.1I Beclin1


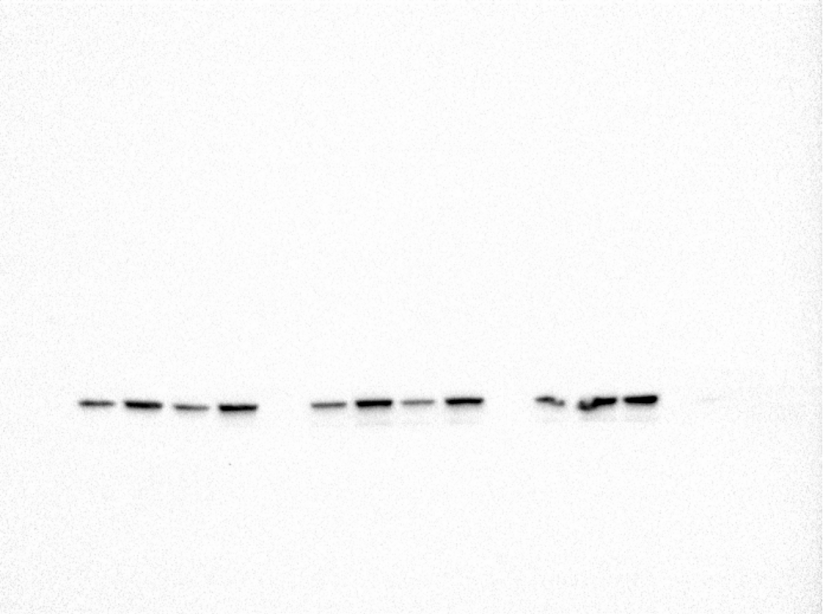


Figure.1I LC3B


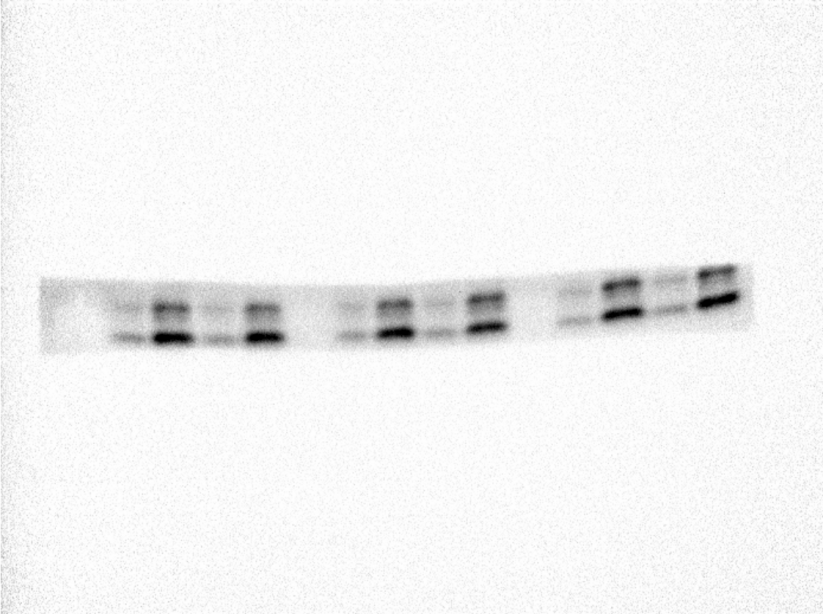


Figure.1I GAPDH


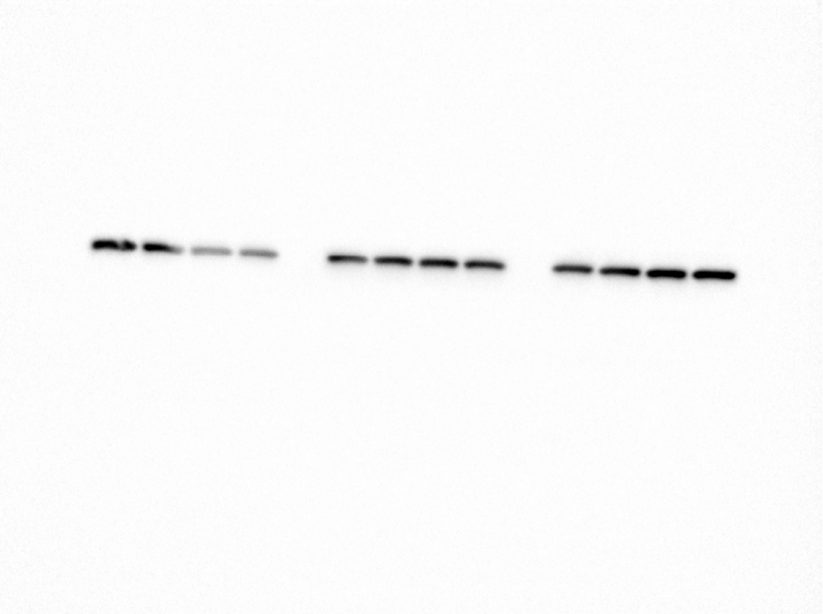


Figure.2F CAPN2


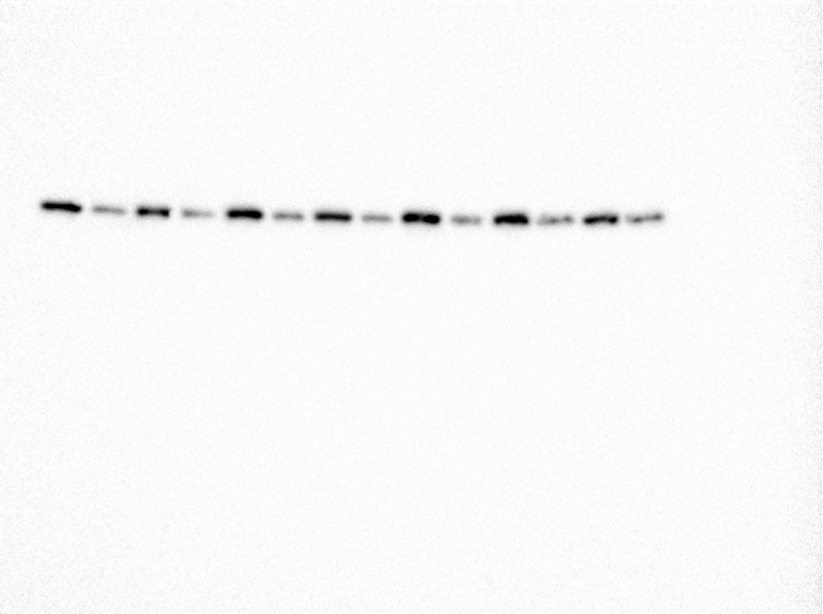


Figure.2F GAPDH


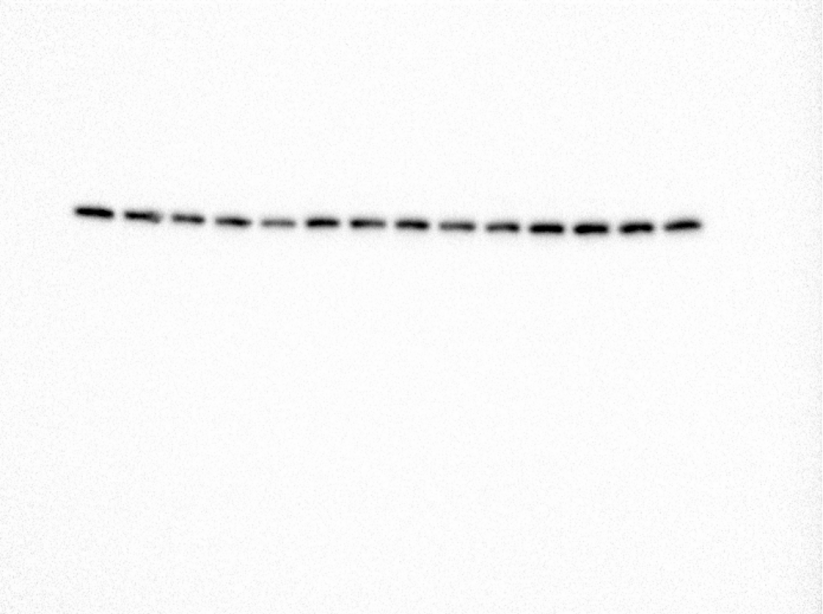


Figure.2G CAPN2


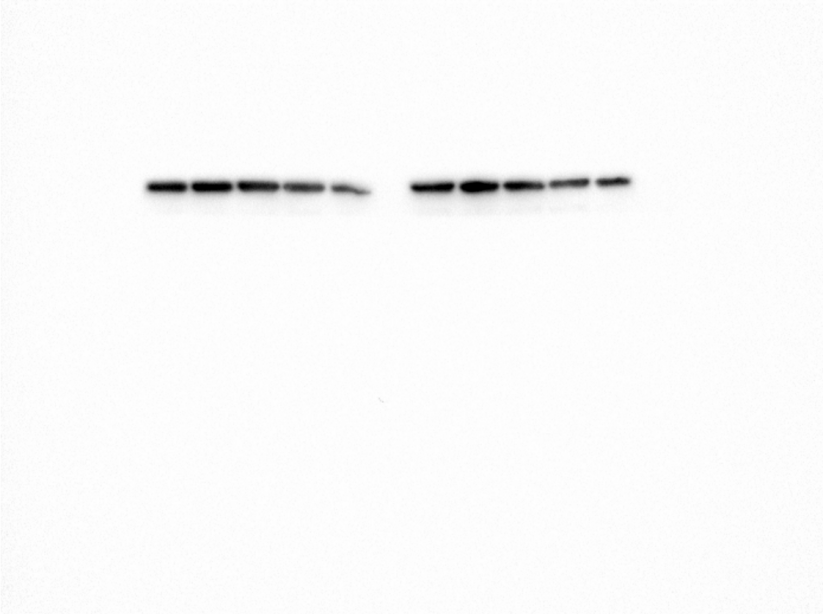


Figure.2G GAPDH


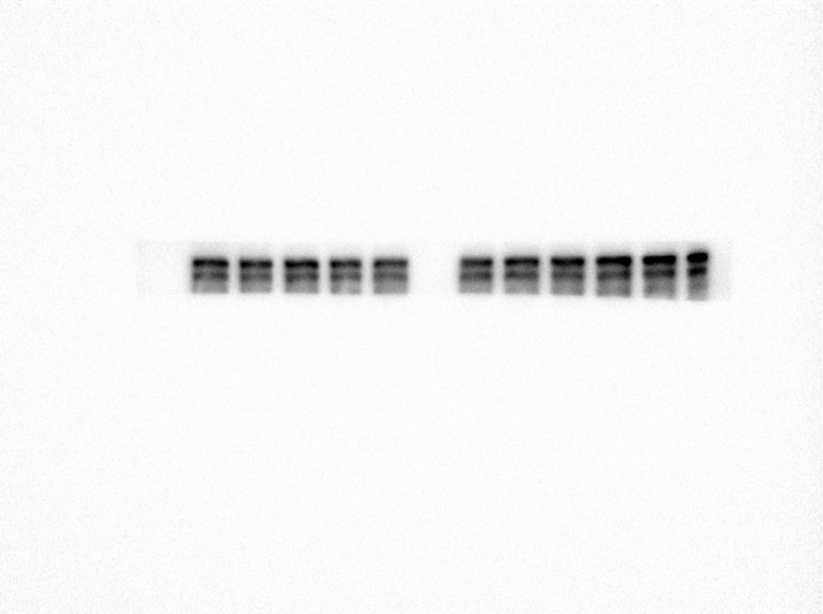


Figure.2H CAPN2


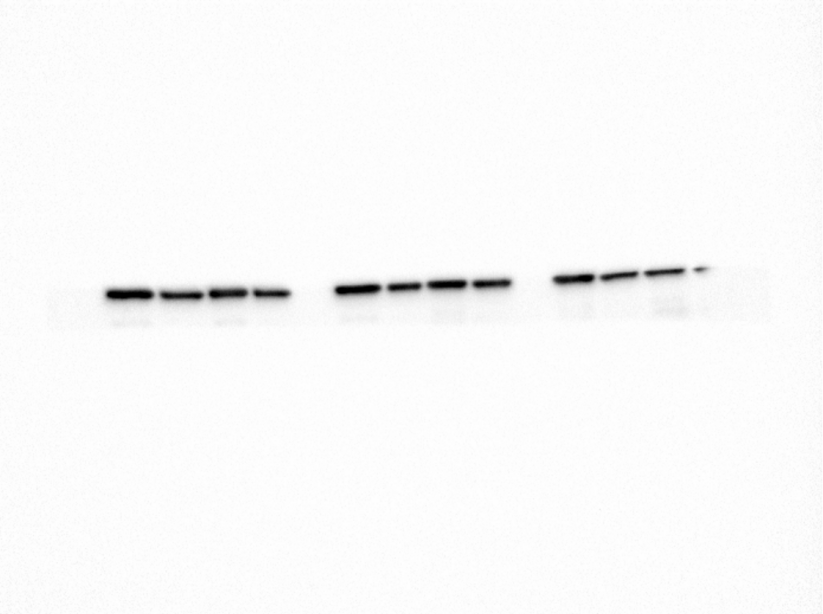


Figure.2H GAPDH


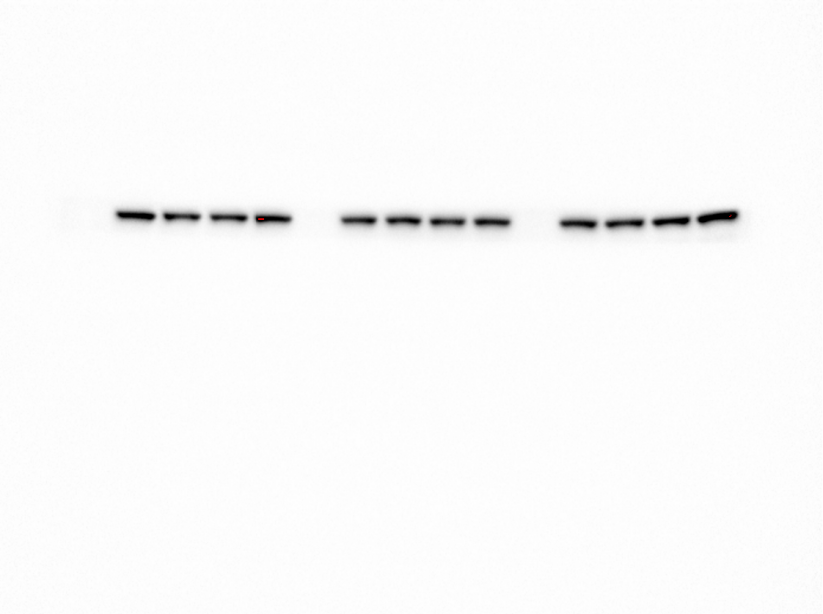


Figure.3B CAPN2


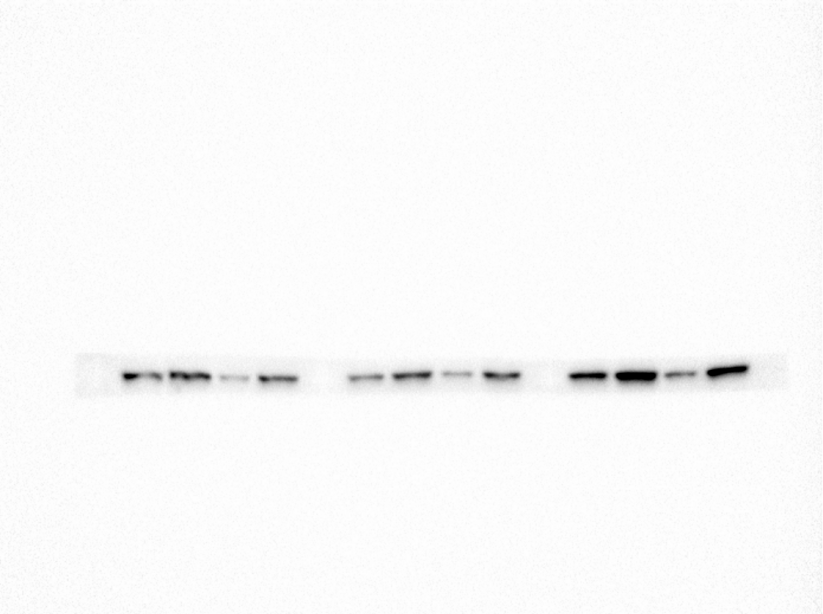


Figure.3B Beclin1


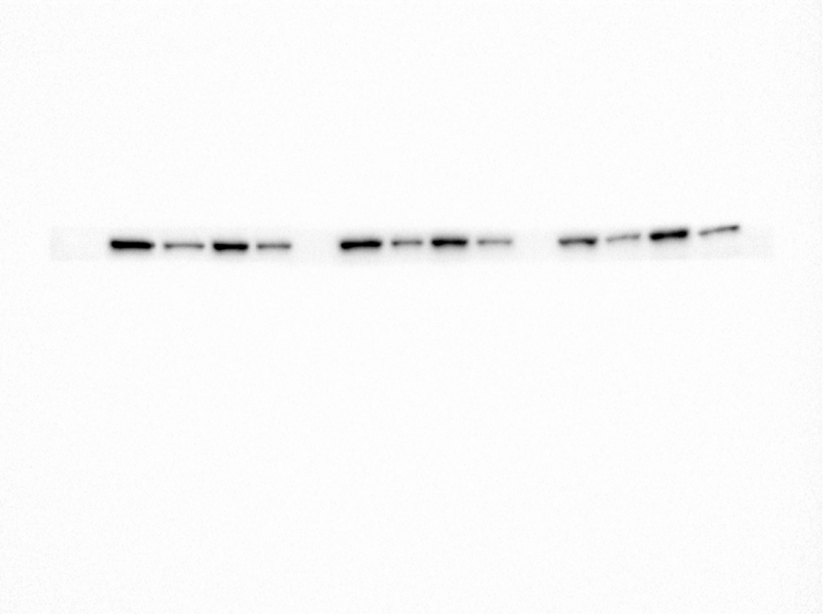


Figure.3B LC3B


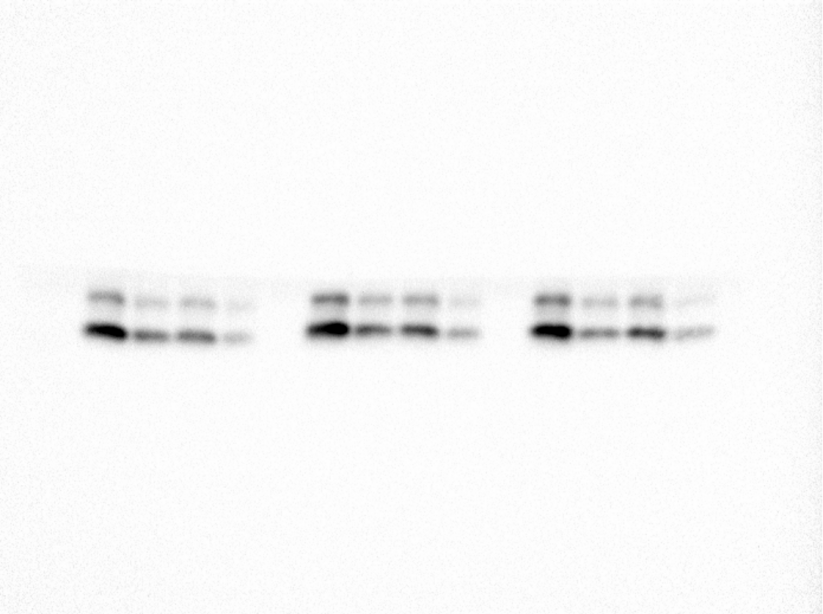


Figure.3B GAPDH


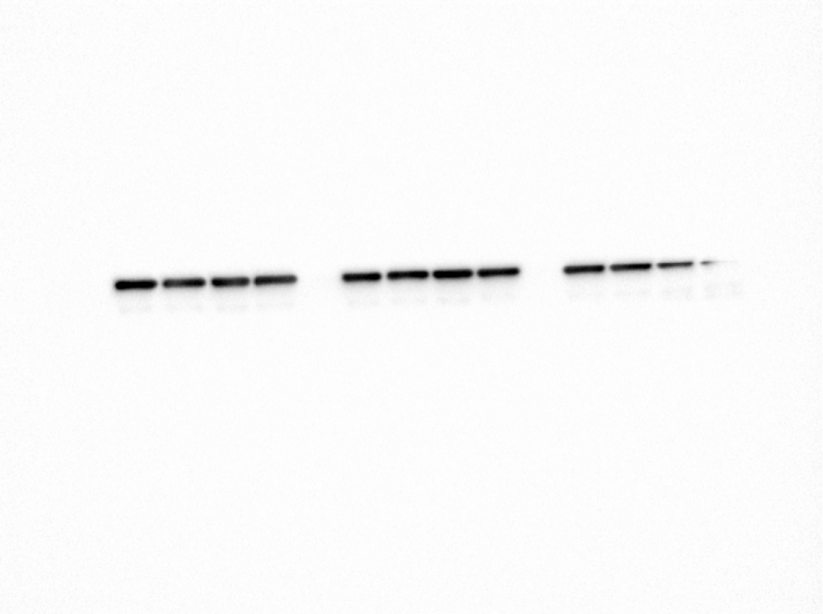


Figure.3G CAPN2


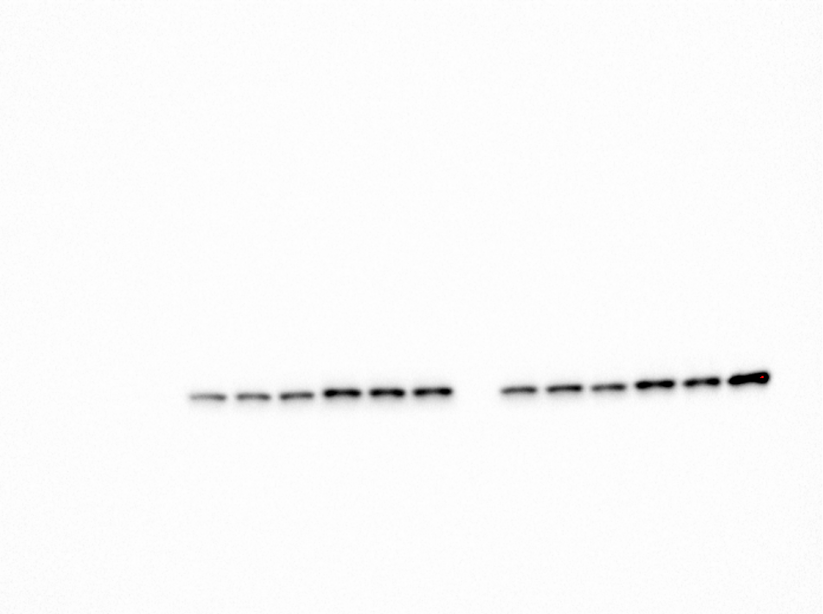


Figure.3G Beclin1


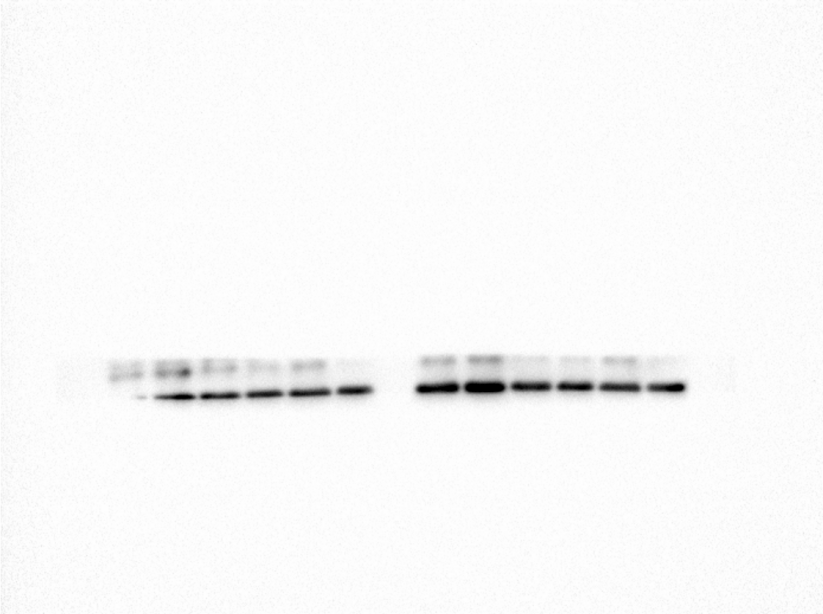


Figure.3G LC3B


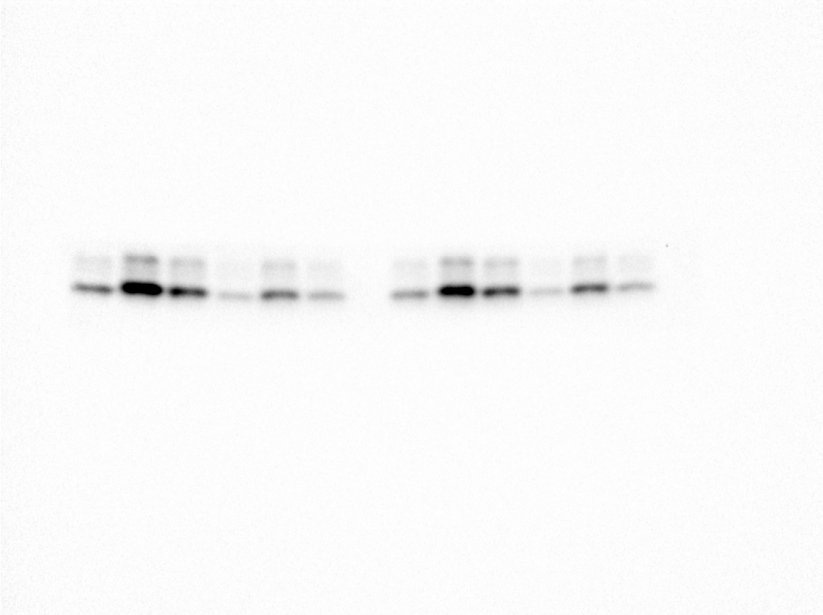


Figure.3G GAPDH


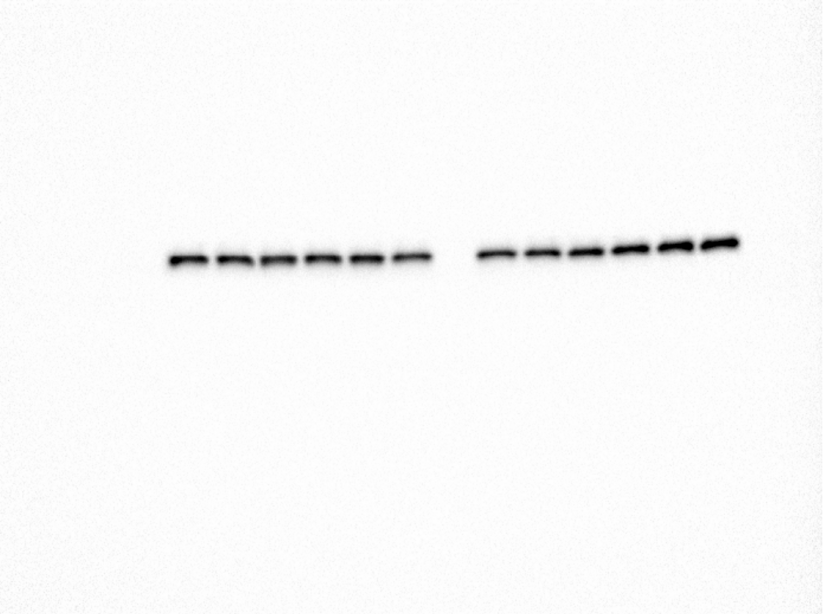


Figure.4D FOXO1


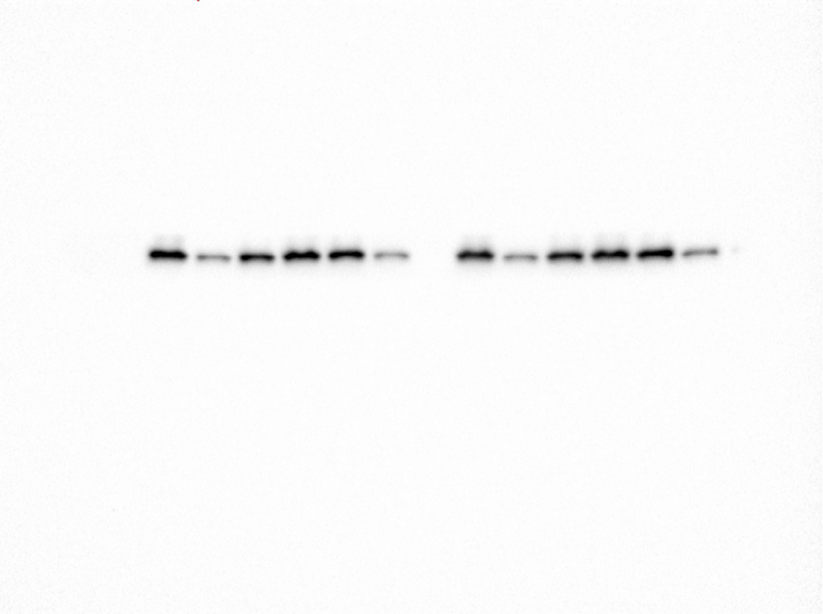


Figure.4D GAPDH


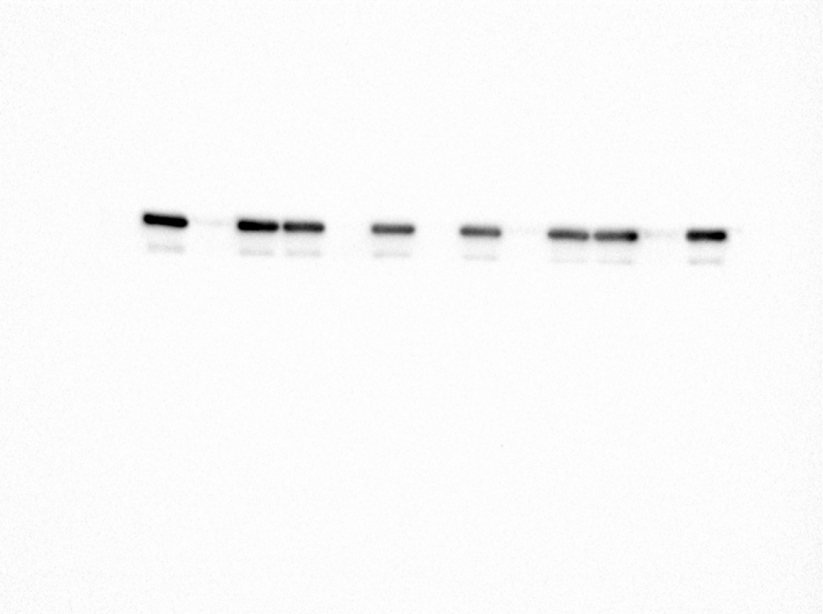


Figure.4D Histon H3


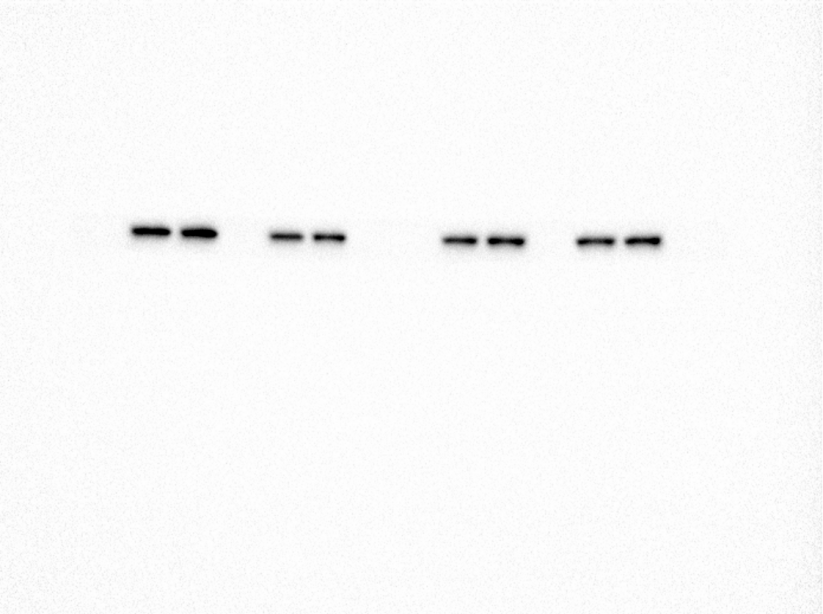


Figure.4F


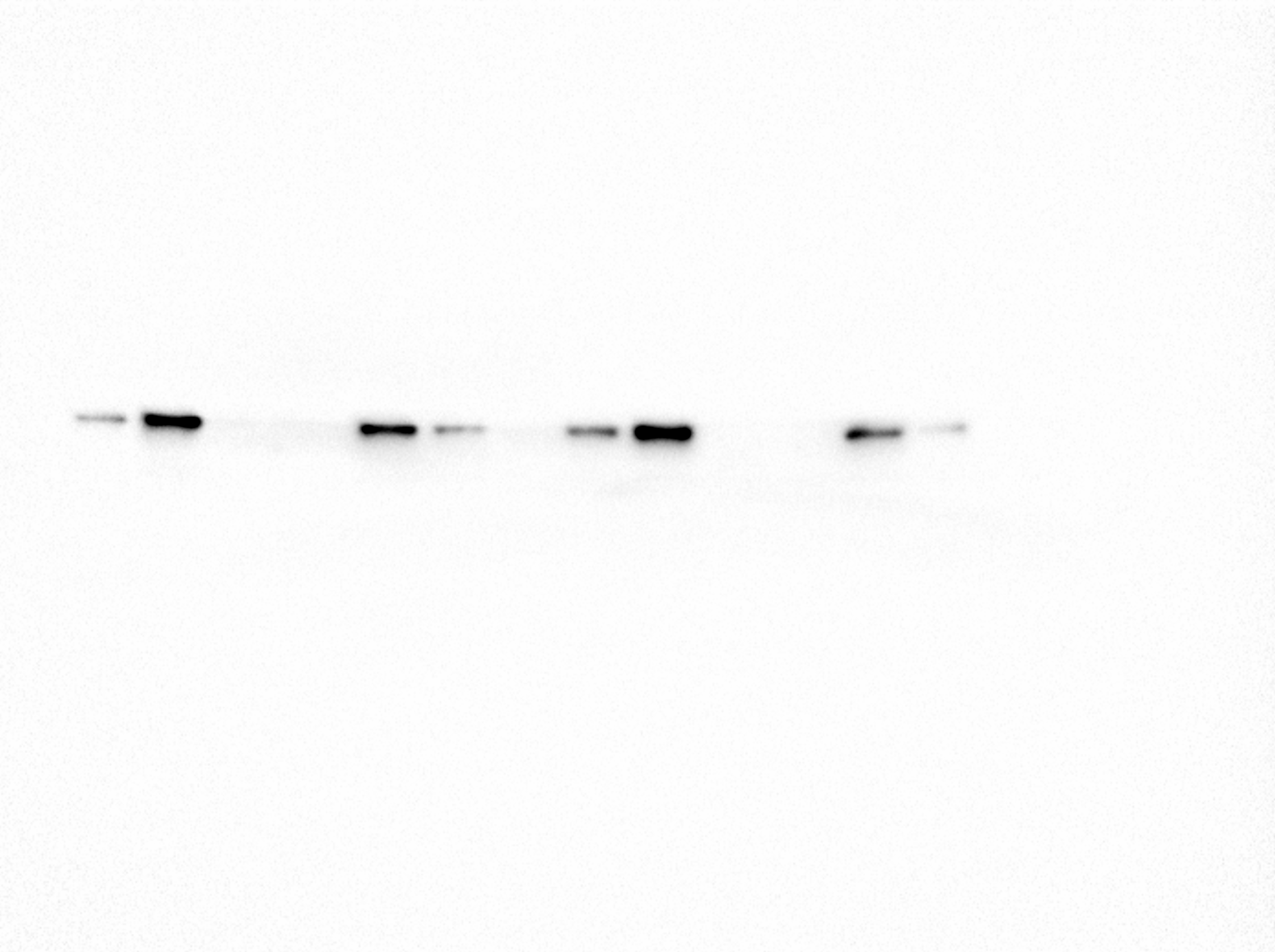

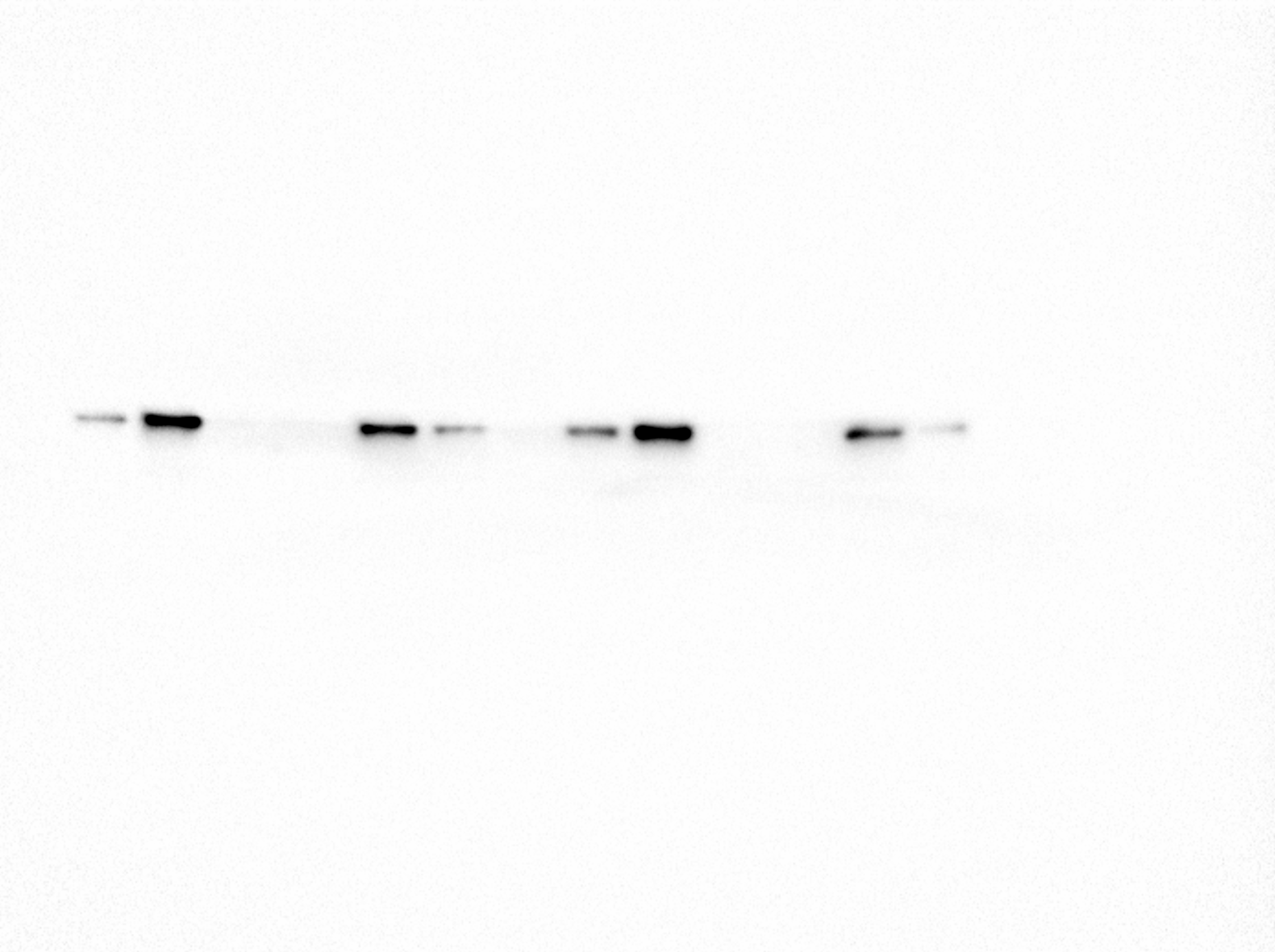


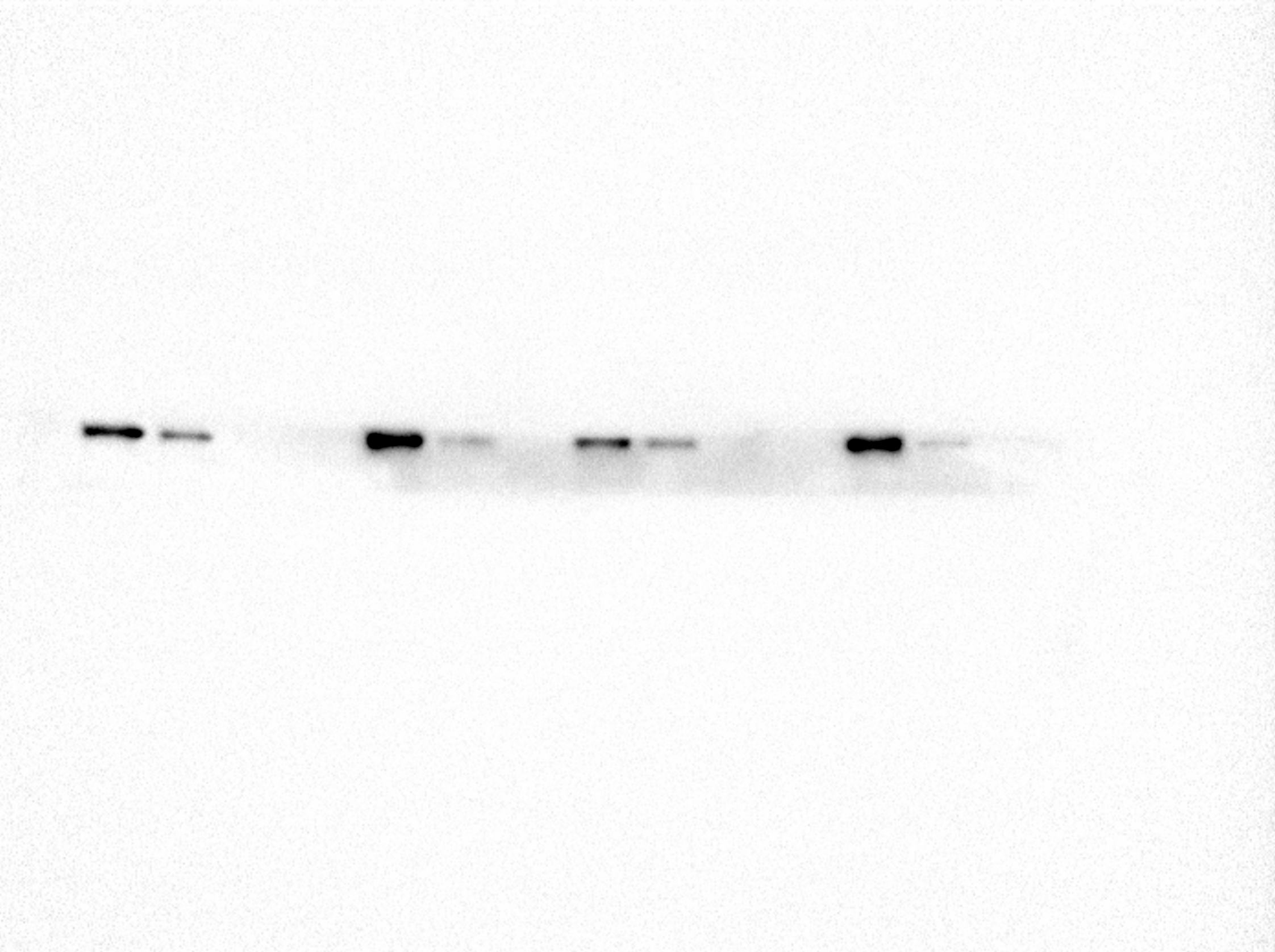

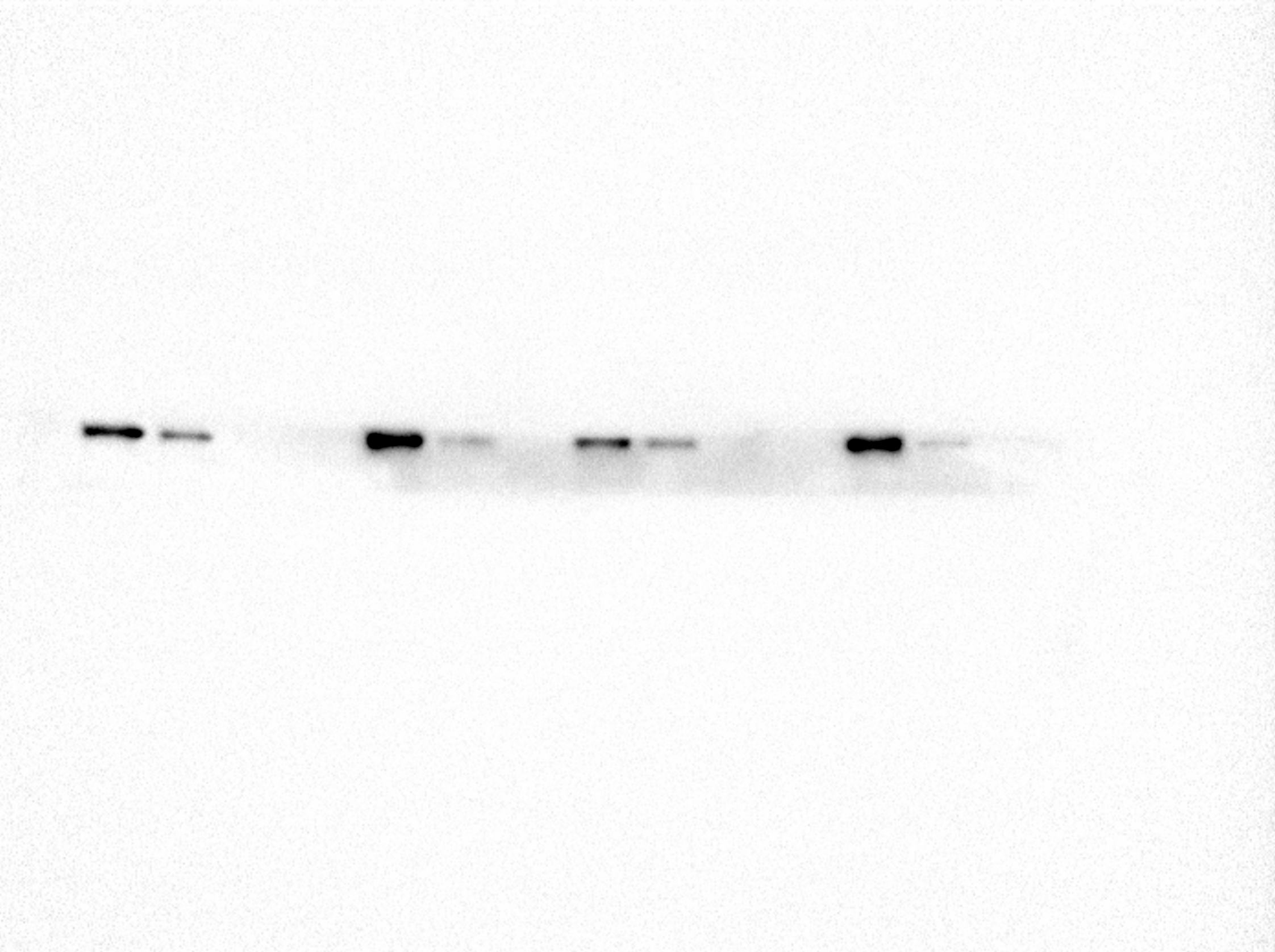
CAPN2


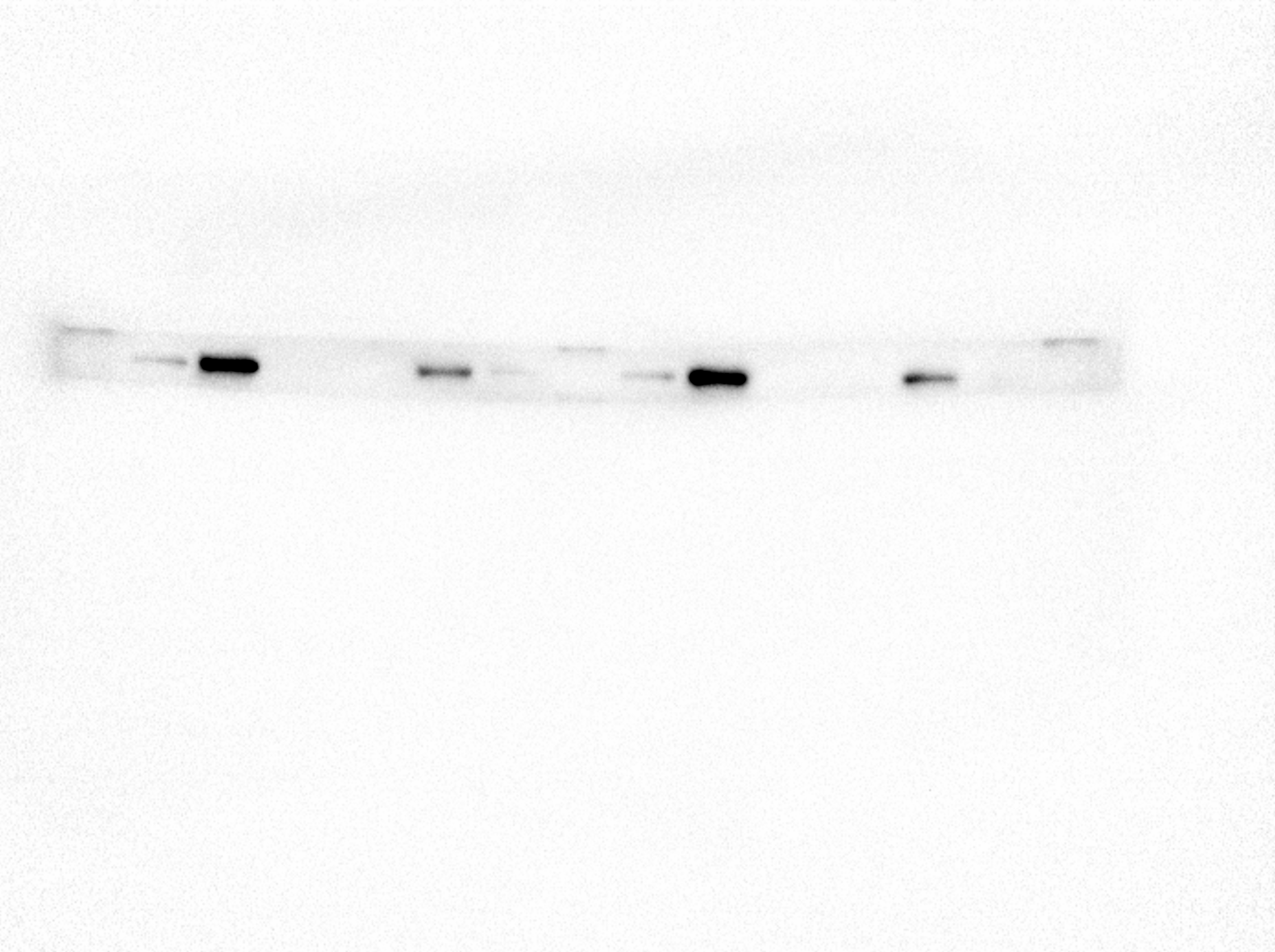

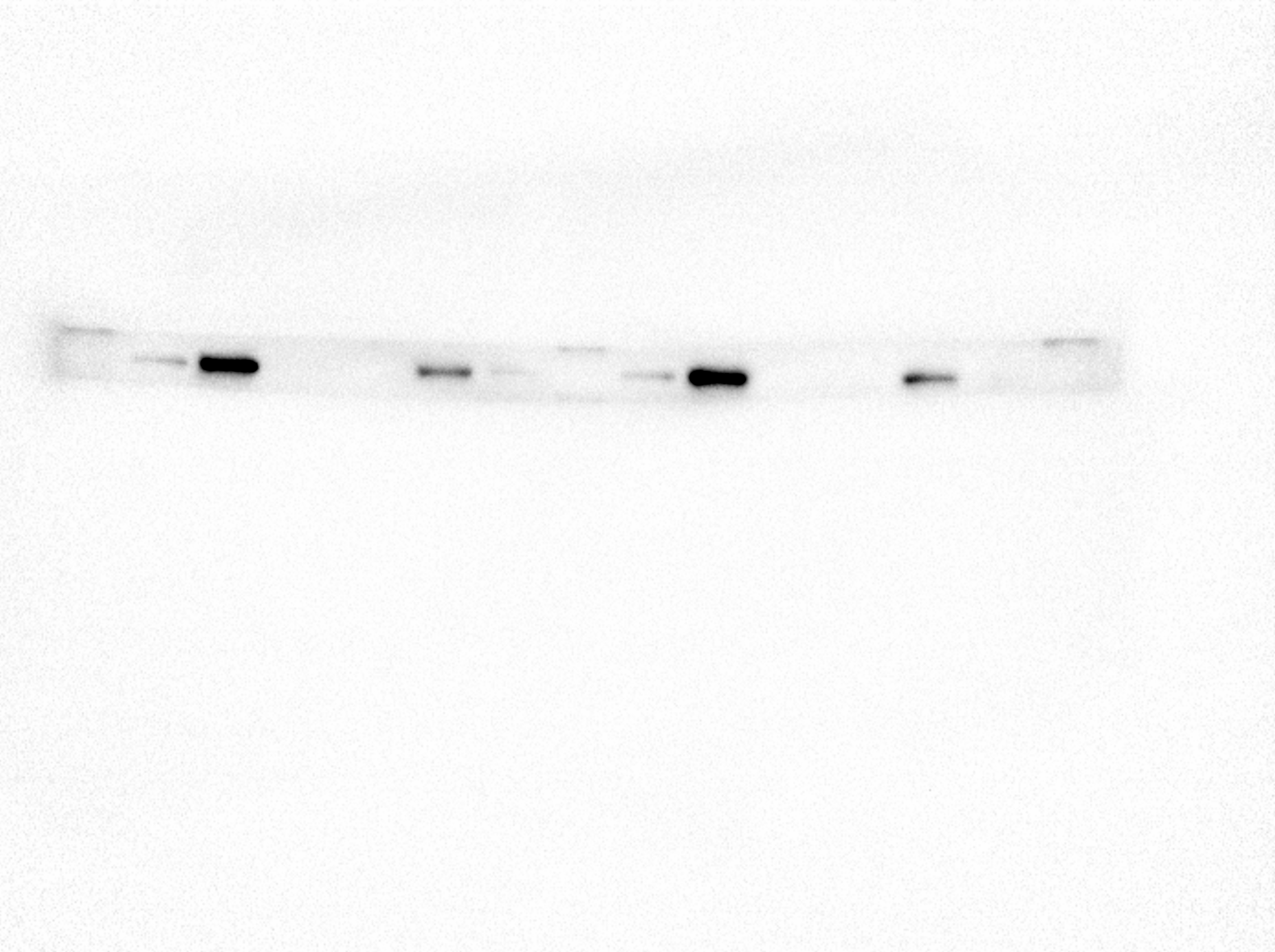

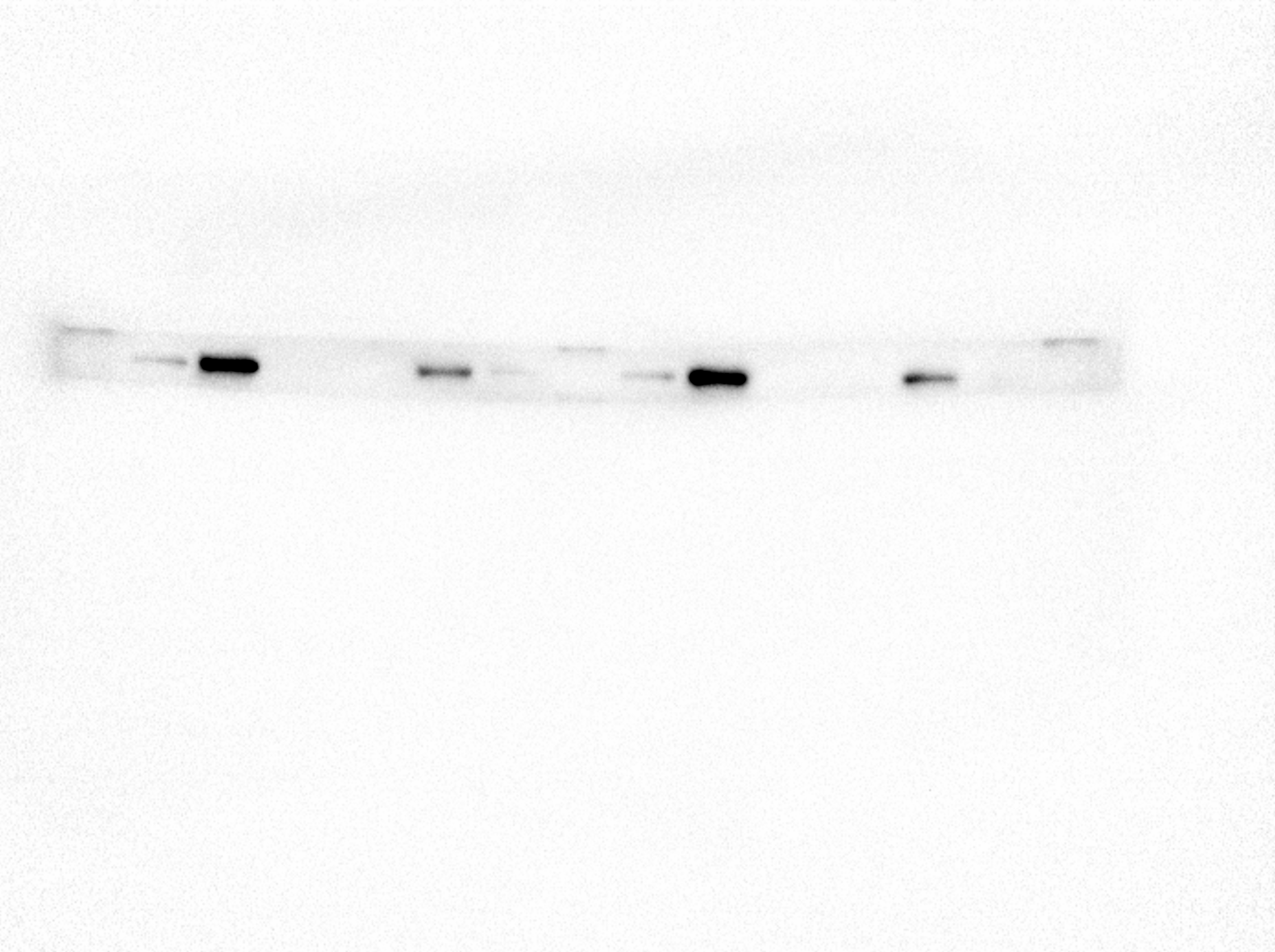

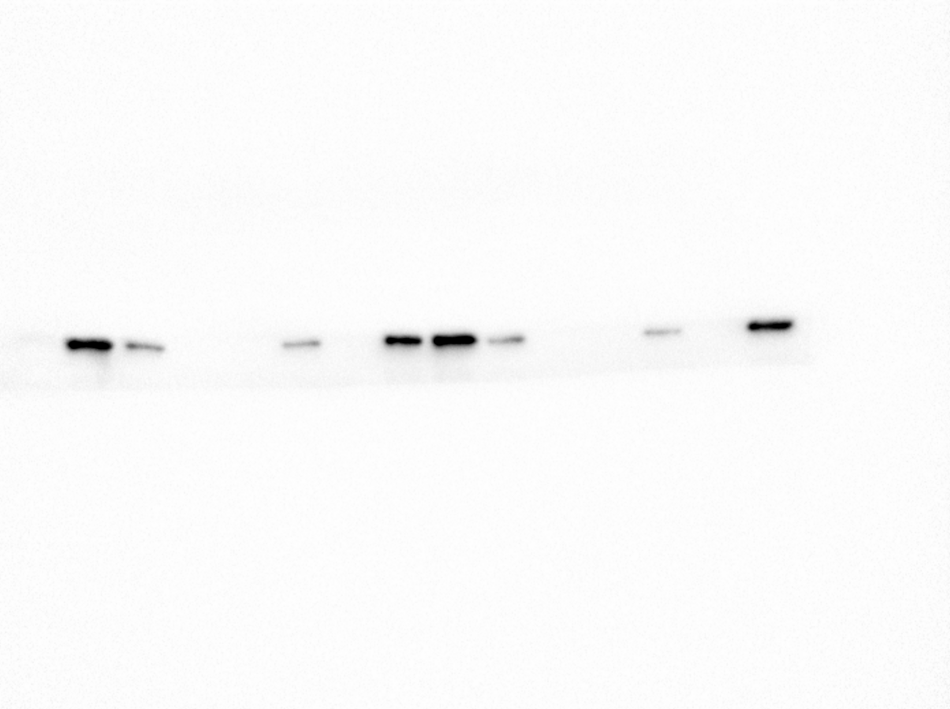


FOXO1


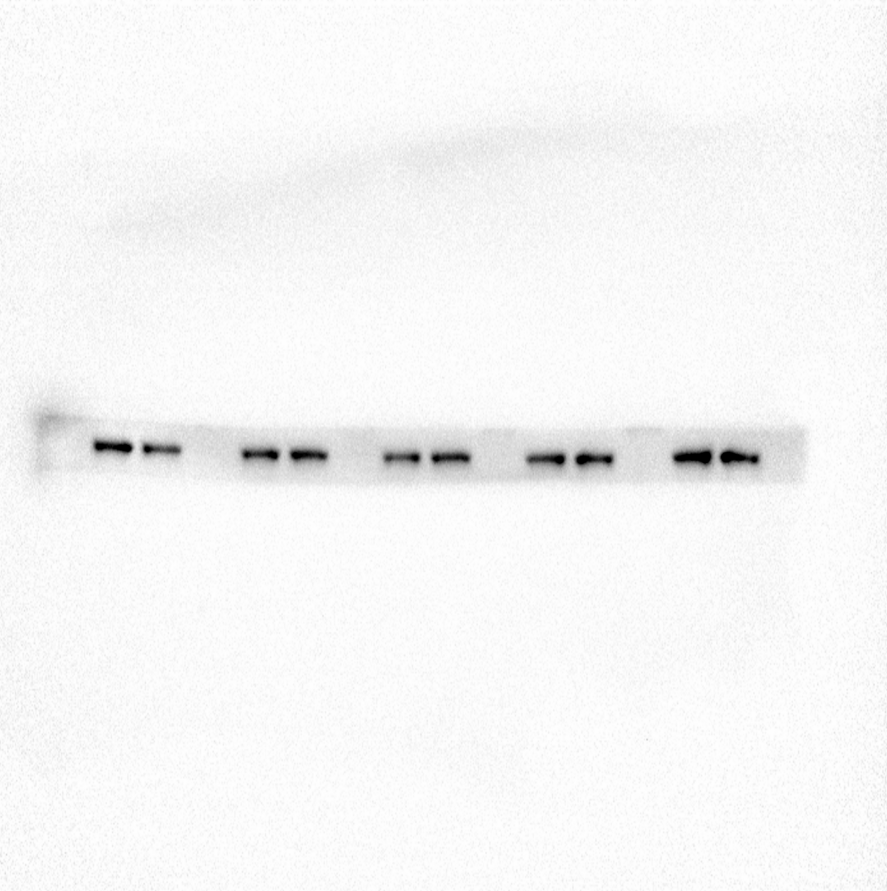

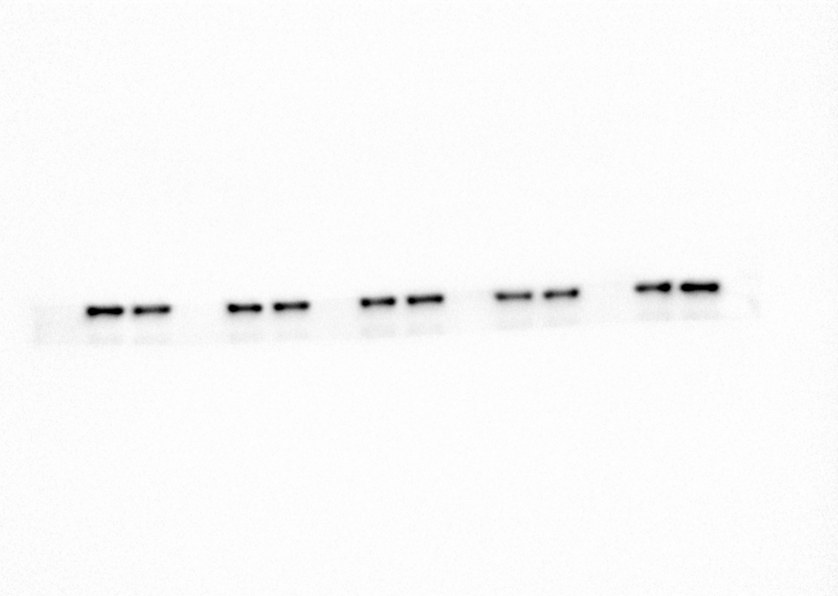


GAPDH

Figure.4H CAPN2


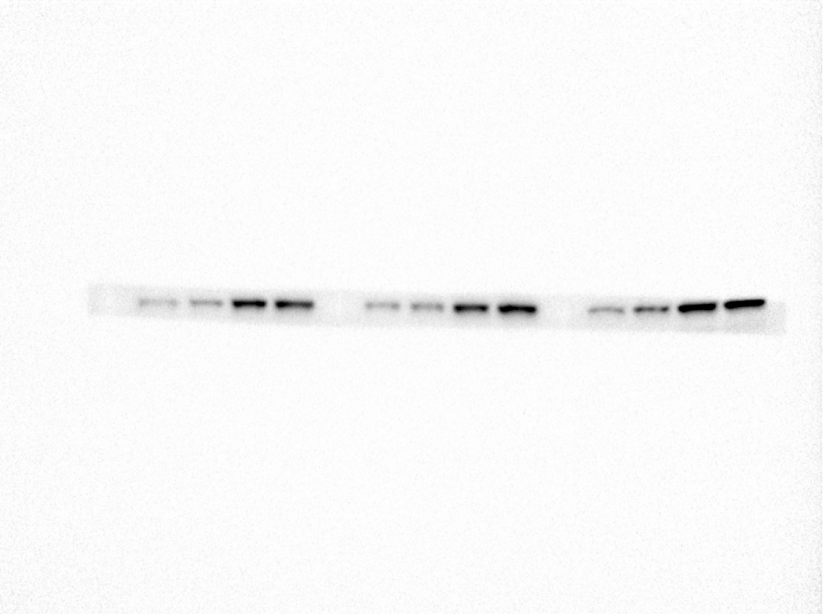


Figure.4H FOXO1


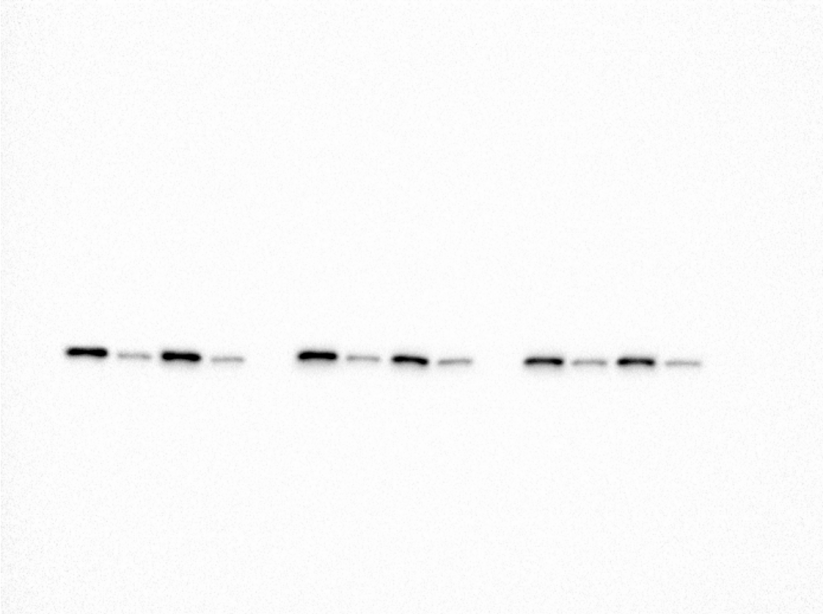


Figure.4H ATG5


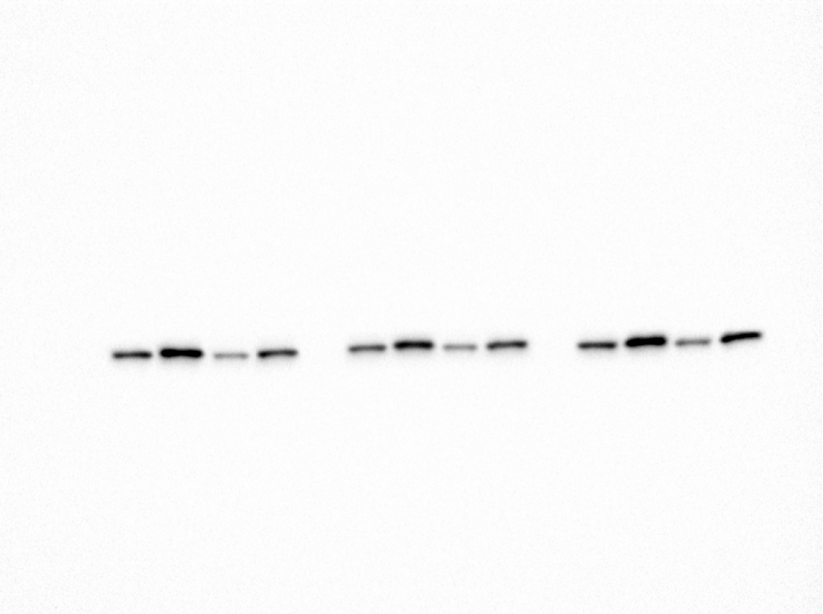


Figure.4G ATG5

Figure.4H P62


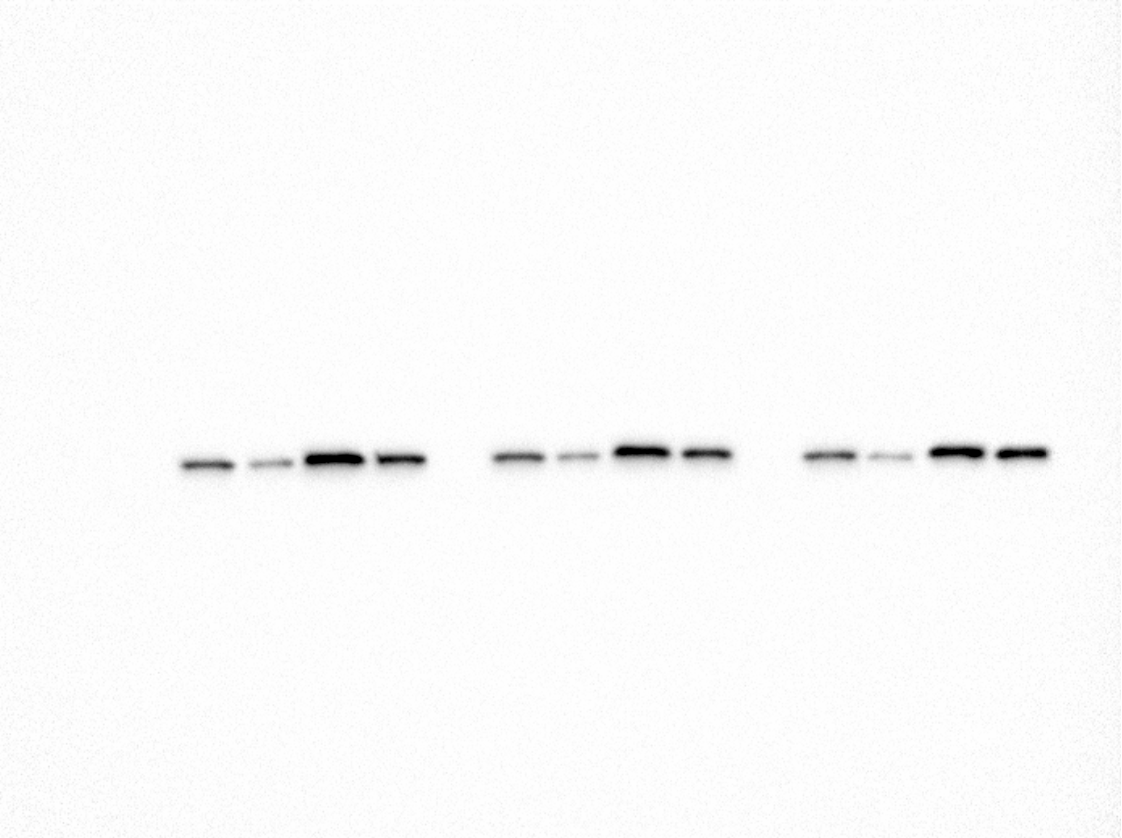


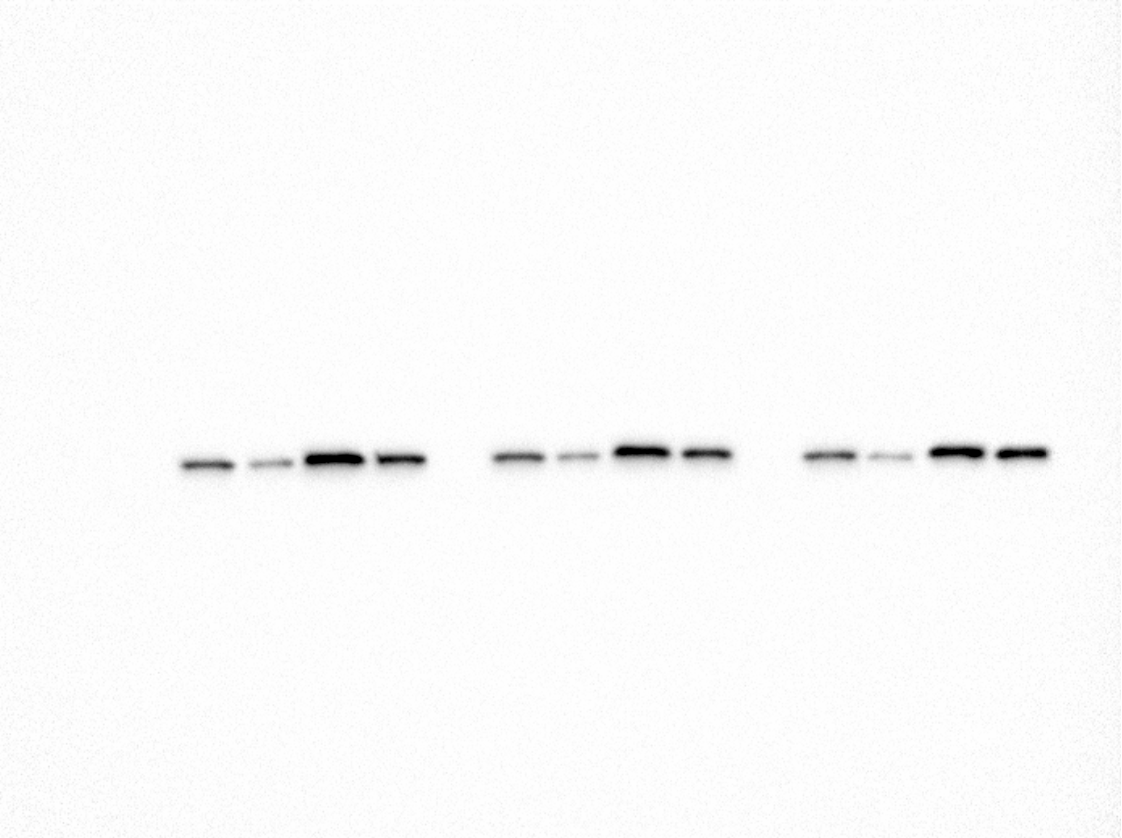


Figure.4H LC3B


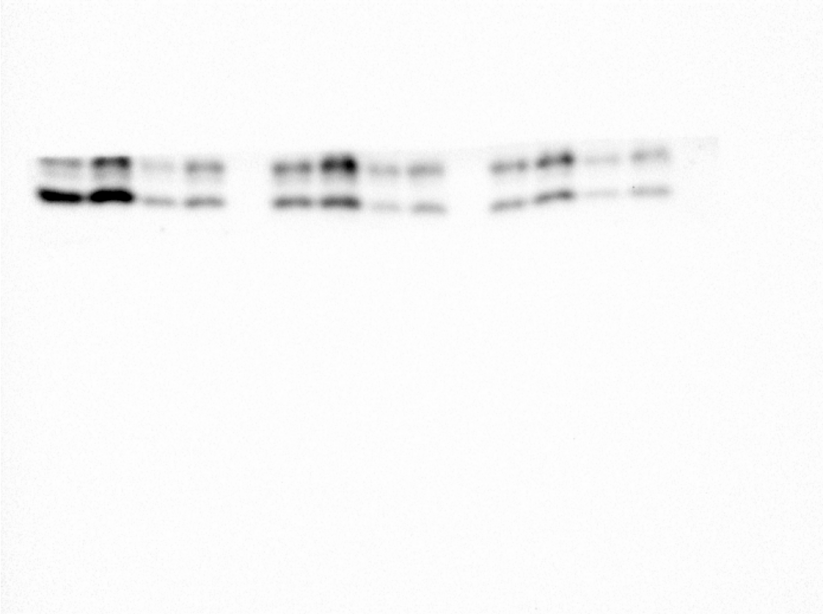


Figure.4G LC3B

Figure.4H GAPDH


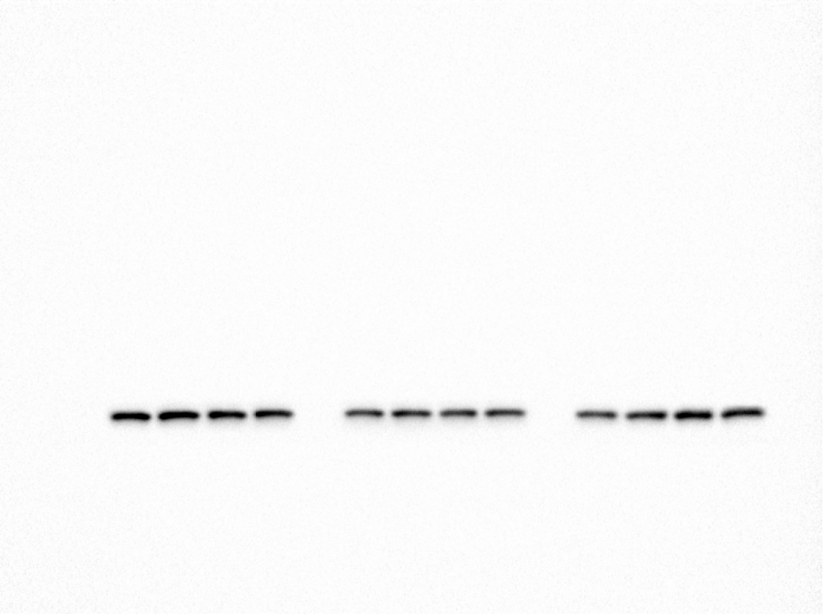


Figure.5I FOXO1


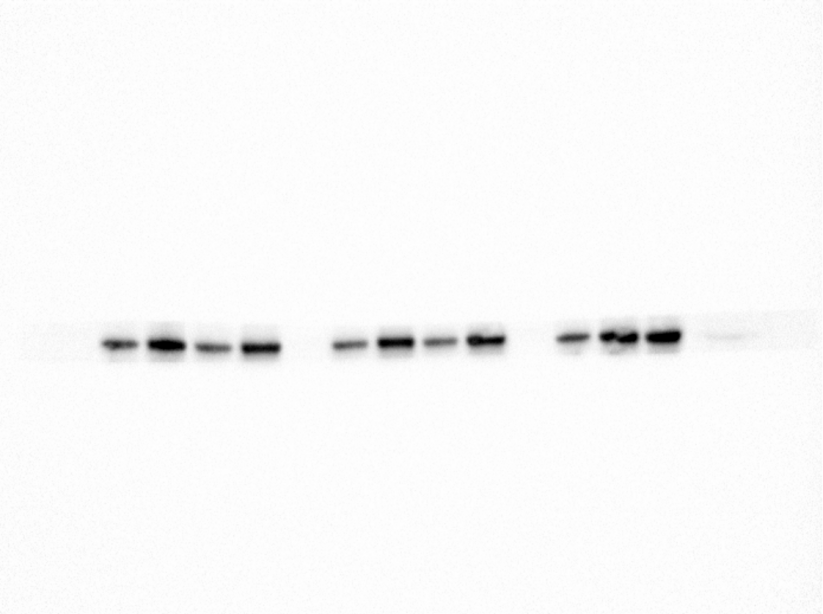


Figure.5I ATG5


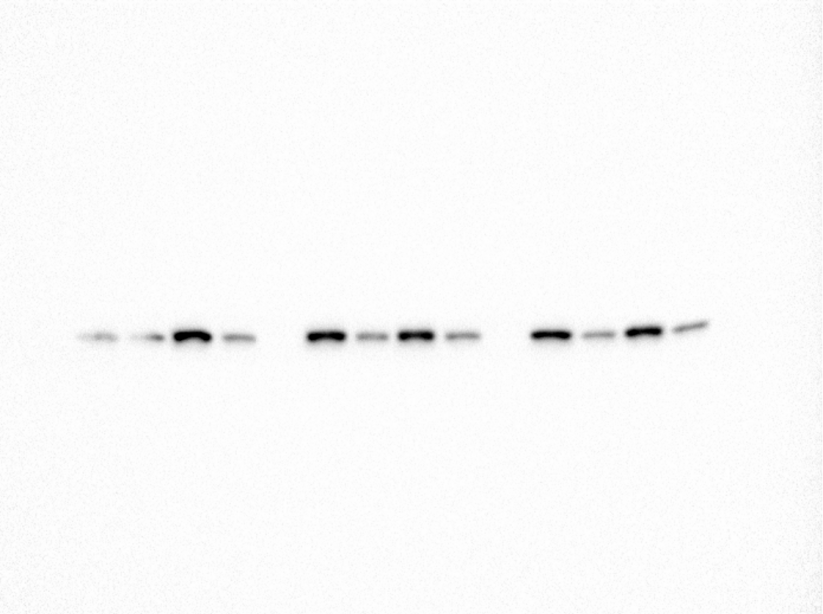


Figure.5I Beclin1


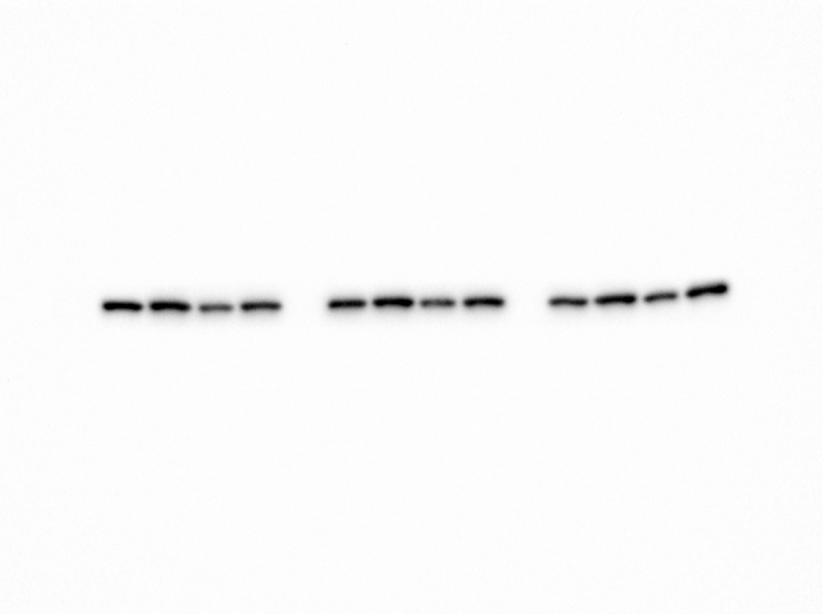


Figure.5I LC3B


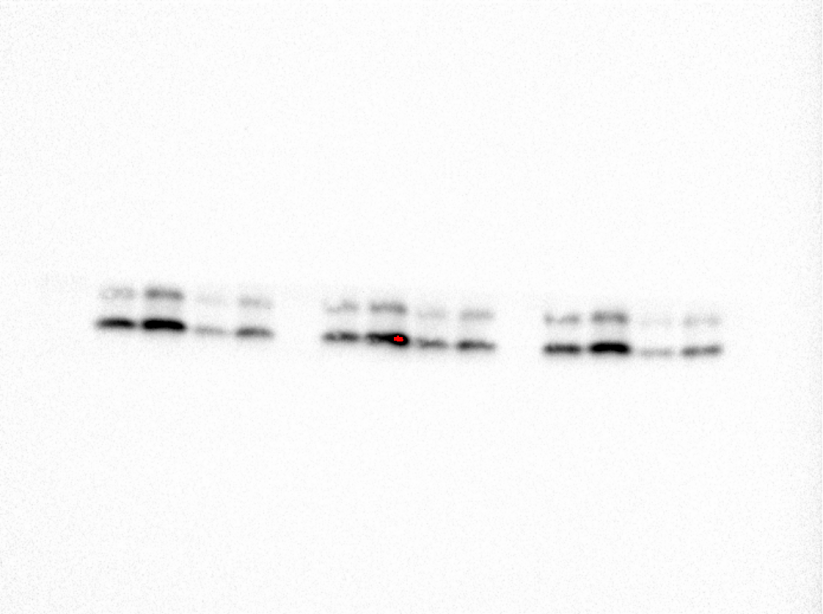


Figure.5I GAPDH


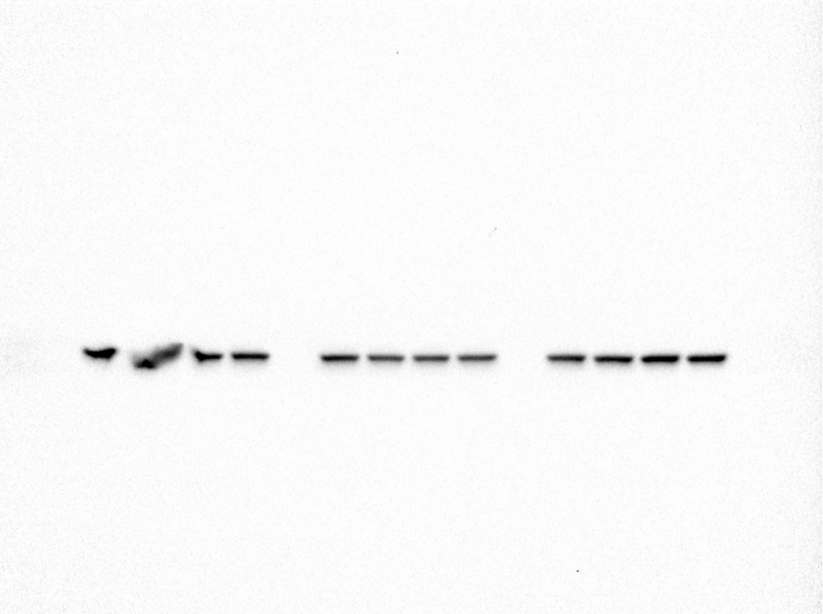


Figure.6F ATF3


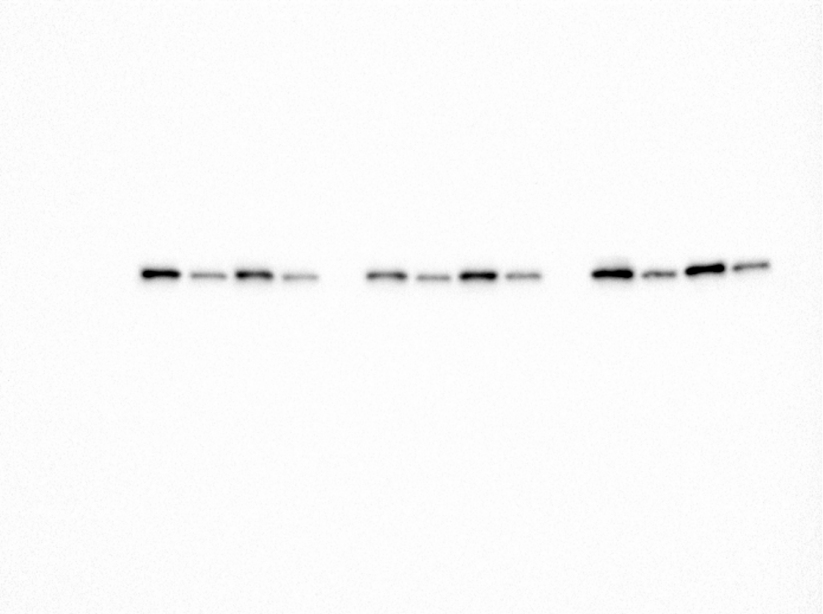


Figure.6F CAPN2


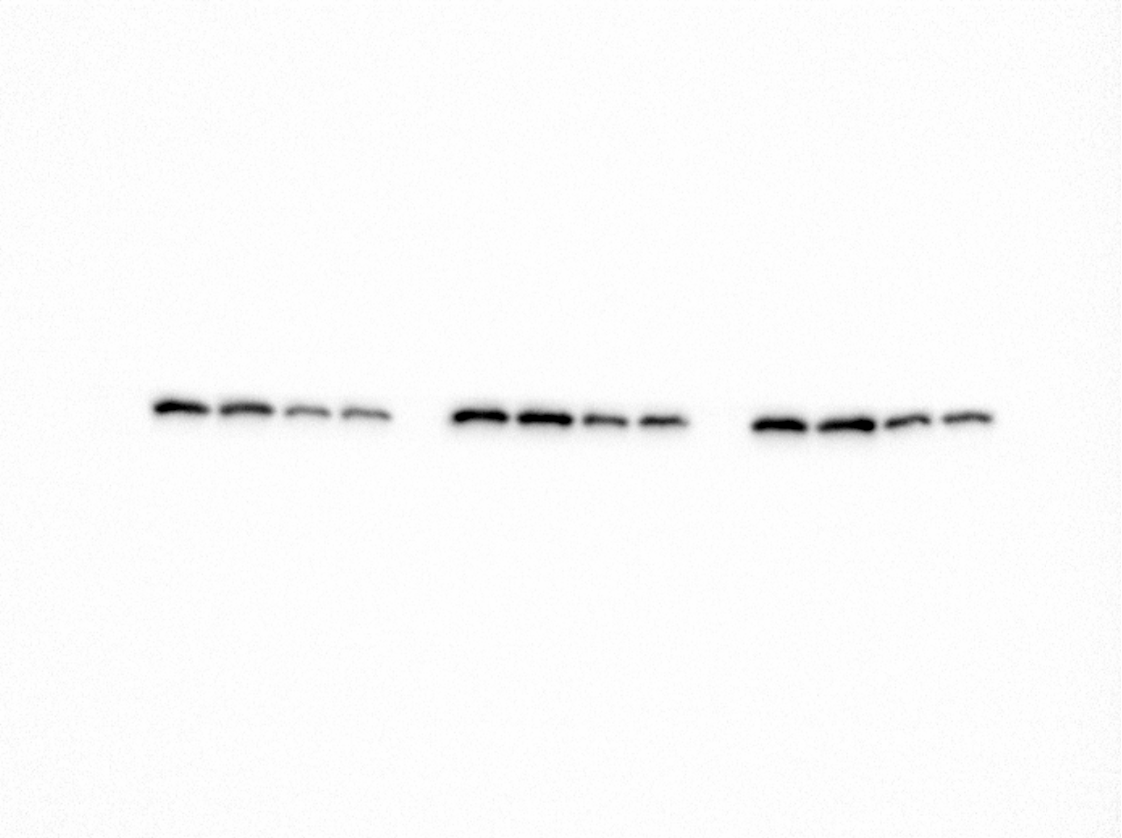

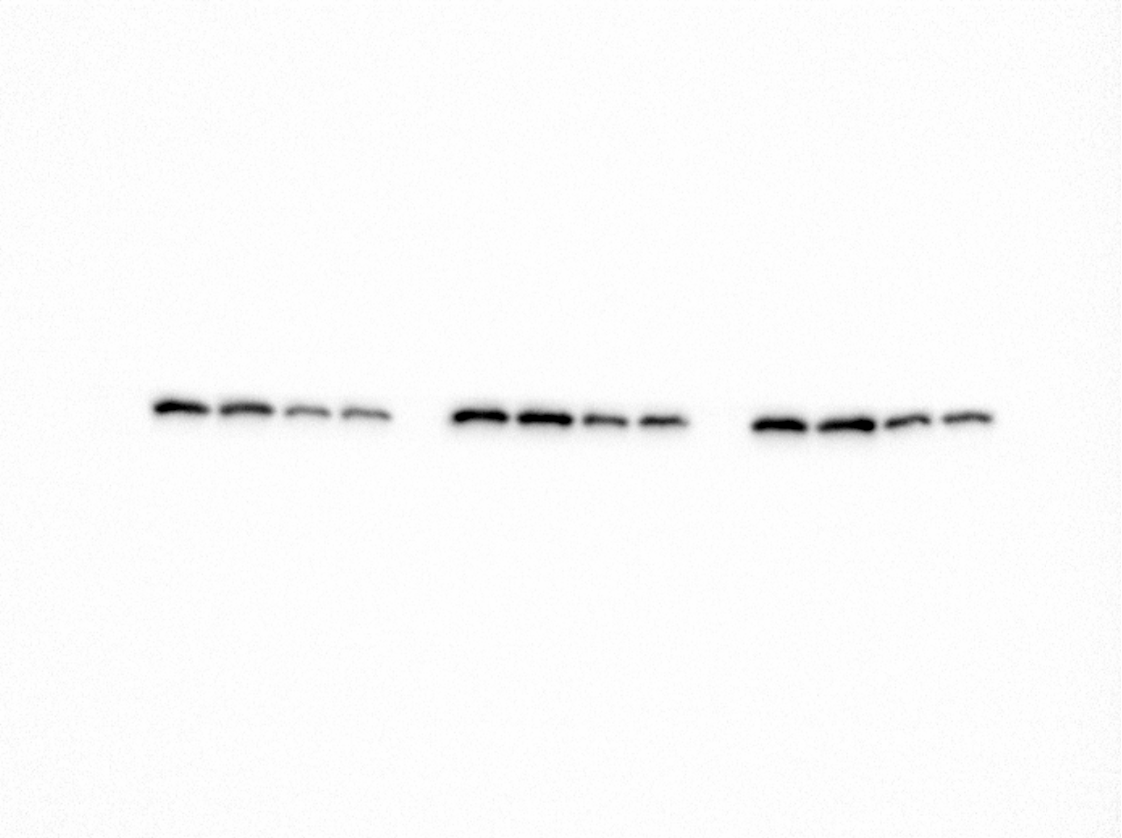


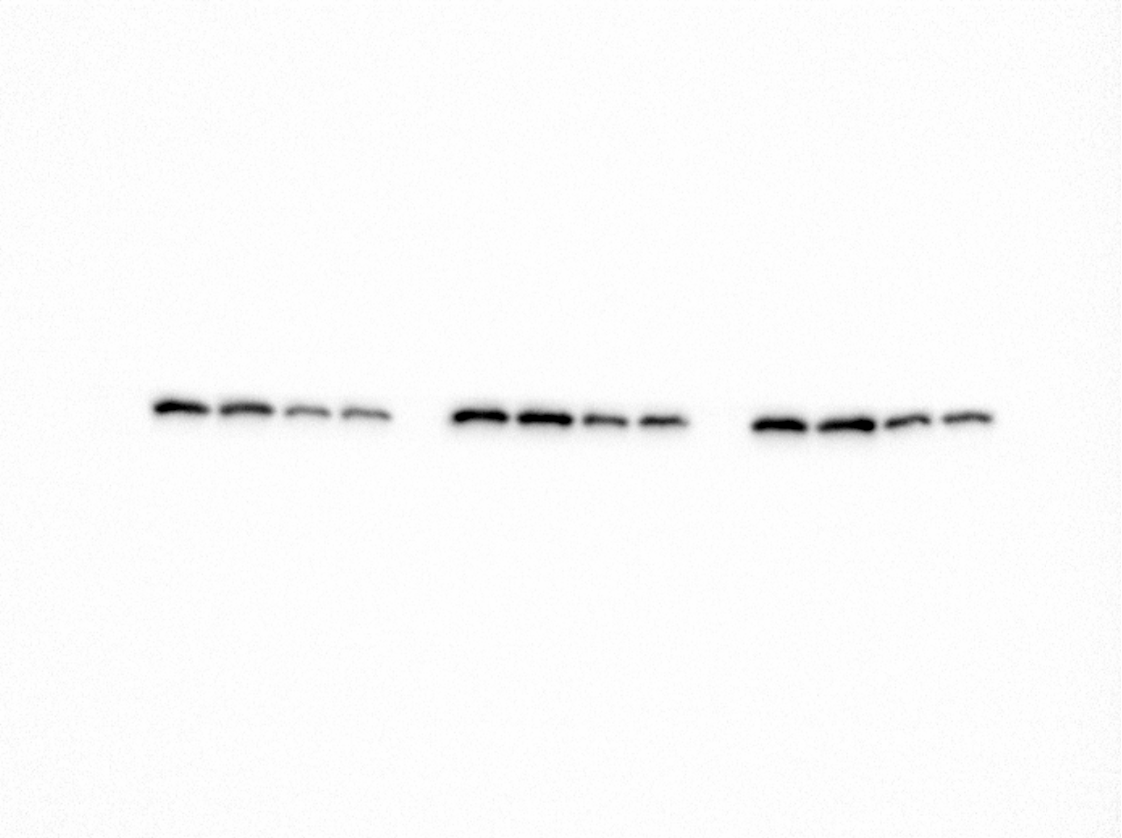


Figure.6F FOXO1


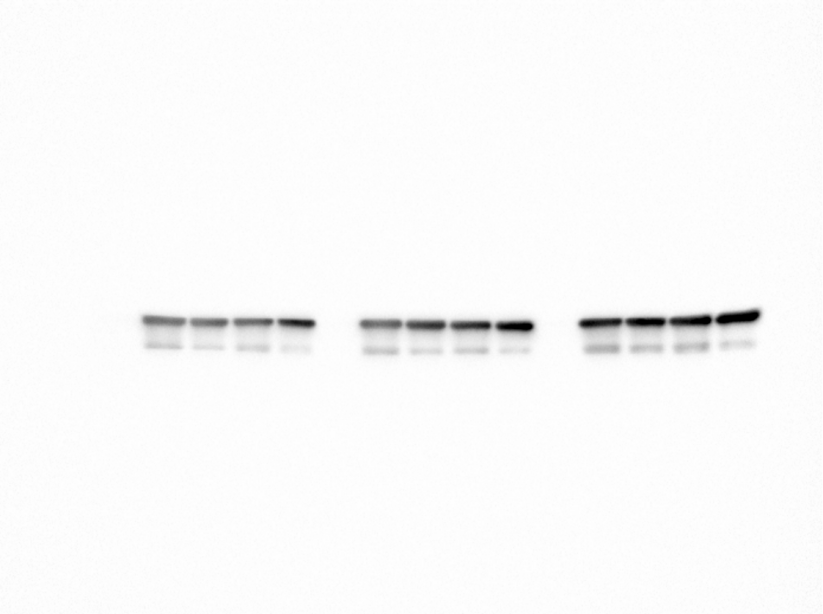


Figure.6F Beclin1


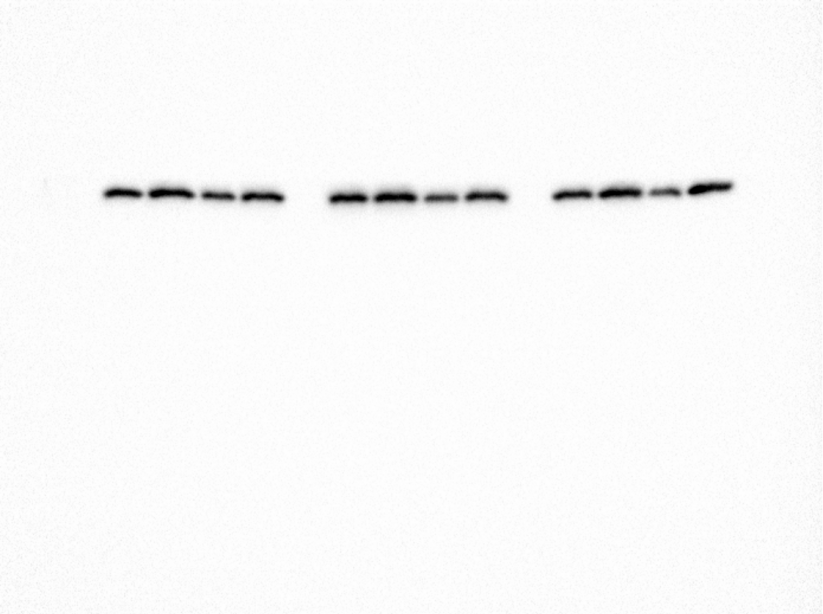


Figure.6F LC3B


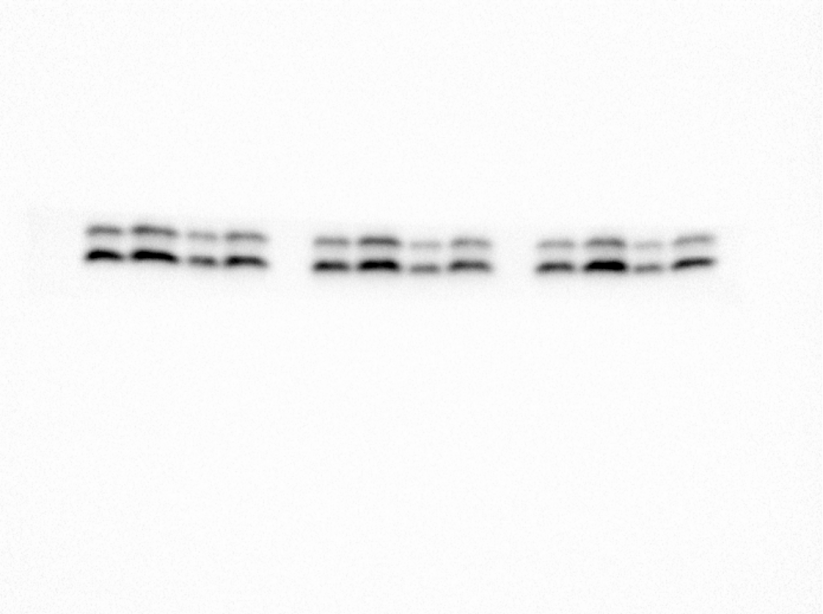


Figure.6F GAPDH


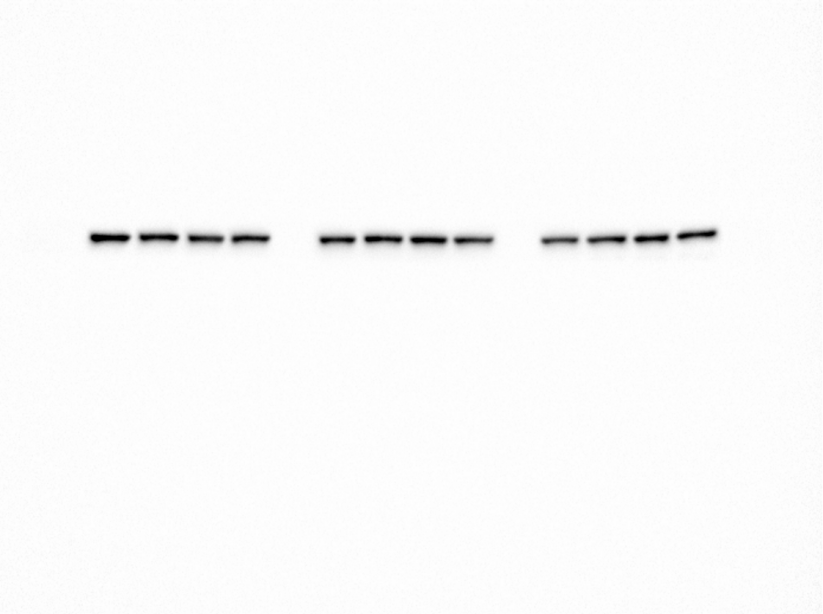

Supplement: Supplementary file 2 — Supplementary Material 2 [file 12967_2024_5335_MOESM2_ESM.docx]
